# Muramyl dipeptide-based analogs as potential anticancer compounds: Strategies to improve selectivity, biocompatibility, and efficiency

**DOI:** 10.3389/fonc.2022.970967

**Published:** 2022-09-27

**Authors:** Eliza Iwicka, Justyna Hajtuch, Krystyna Dzierzbicka, Iwona Inkielewicz-Stepniak

**Affiliations:** ^1^ Department of Pharmaceutical Pathophysiology, Medical University of Gdansk, Gdansk, Poland; ^2^ Department of Organic Chemistry, Gdansk University of Technology, Gdansk, Poland

**Keywords:** muramyl dipeptide, muramyl dipeptide analogs, anticancer compounds, anticancer activity, NOD2 receptor, adjuvant therapy

## Abstract

According to the WHO, cancer is the second leading cause of death in the world. This is an important global problem and a major challenge for researchers who have been trying to find an effective anticancer therapy. A large number of newly discovered compounds do not exert selective cytotoxic activity against tumorigenic cells and have too many side effects. Therefore, research on muramyl dipeptide (MDP) analogs has attracted interest due to the urgency for finding more efficient and safe treatments for oncological patients. MDP is a ligand of the cytosolic nucleotide-binding oligomerization domain 2 receptor (NOD2). This molecule is basic structural unit that is responsible for the immune activity of peptidoglycans and exhibits many features that are important for modern medicine. NOD2 is a component of the innate immune system and represents a promising target for enhancing the innate immune response as well as the immune response against cancer cells. For this reason, MDP and its analogs have been widely used for many years not only in the treatment of immunodeficiency diseases but also as adjuvants to support improved vaccine delivery, including for cancer treatment. Unfortunately, in most cases, both the MDP molecule and its synthesized analogs prove to be too pyrogenic and cause serious side effects during their use, which consequently exclude them from direct clinical application. Therefore, intensive research is underway to find analogs of the MDP molecule that will have better biocompatibility and greater effectiveness as anticancer agents and for adjuvant therapy. In this paper, we review the MDP analogs discovered in the last 10 years that show promise for antitumor therapy. The first part of the paper compiles the achievements in the field of anticancer vaccine adjuvant research, which is followed by a description of MDP analogs that exhibit promising anticancer and antiproliferative activity and their structural changes compared to the original MDP molecule.

## Introduction

Peptidoglycan (PGN) is one of the main and extremely important components of bacterial cell walls in both gram-positive and gram-negative bacteria. It has a crystalline structure, which is due to the presence of linear β-1,4 glycan chains linked by two amino sugars: *N*-acetylmuramic acid (Mur*N*Ac) and *N*-acetylglucosamine (Glc*N*Ac) (1, 2). MDP, or *N*-acetylmuramyl-L-alanyl-D-isoglutamine, is the basic and fundamental building unit that is a part of PGNs and responsible for the structure of the bacterial cell wall and the immune response of the same (3).

**Figure 1:**
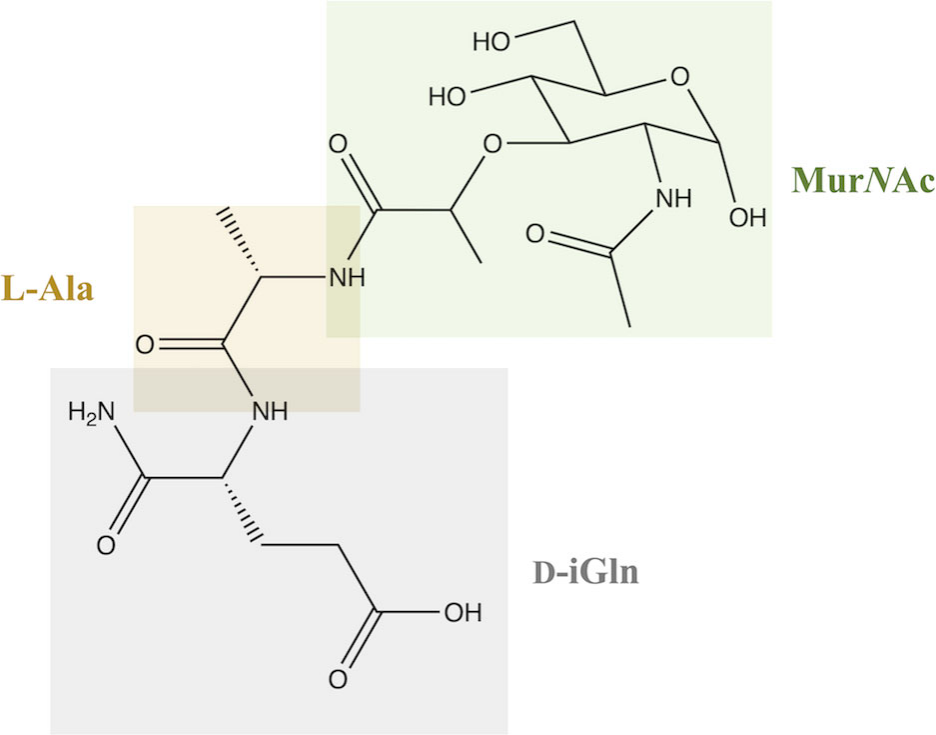
Structure of MDP.

In terms of structure, MDP consists of Mur*N*Ac and two amino acids, namely L-Ala and D-iGln (**Figure 1**). It is a glycopeptide that shows stimulatory effects on many monocyte and macrophage functions, such as pinocytosis, phagocytosis, chemotaxis, and bactericidal and antitumor activity.

It has been found that one of the most interesting and medically relevant biological activities of MDP is the induction of pro-inflammatory release cytokines from human monocytes, which occurs *via* toll-like receptor (TLR) and NOD2-dependent signaling pathways (4). The oligomerization domains that bind the nucleotides NOD1 and NOD2 are involved in the recognition of PGN in the cytosol of cells (5). They are intracellularly related sensors of PGN and belong to the NOD-like receptor family of innate immunity proteins. They play an essential role in controlling inflammation and defending the host against infection (6).

The innate immune system is activated when biological motifs that resemble pathogenic molecular patterns appear and are detected (5). The NOD1 receptor is activated by γ-D-glutamyl-meso-diaminopimelic acid, which is mainly found in gram-negative bacteria, and, in turn, the NOD2 pathogen recognition receptor molecule is a PGN sensor that recognizes the MDP generally present in all bacterial PGNs. However, it can only be activated by MDPs that have an intact Mur*N*Ac ring structure, and the sugar must be attached to the dipeptide or tripeptide (Mur*N*Ac-L-Ala-D-Glu motif) (6).

**Figure 2:**
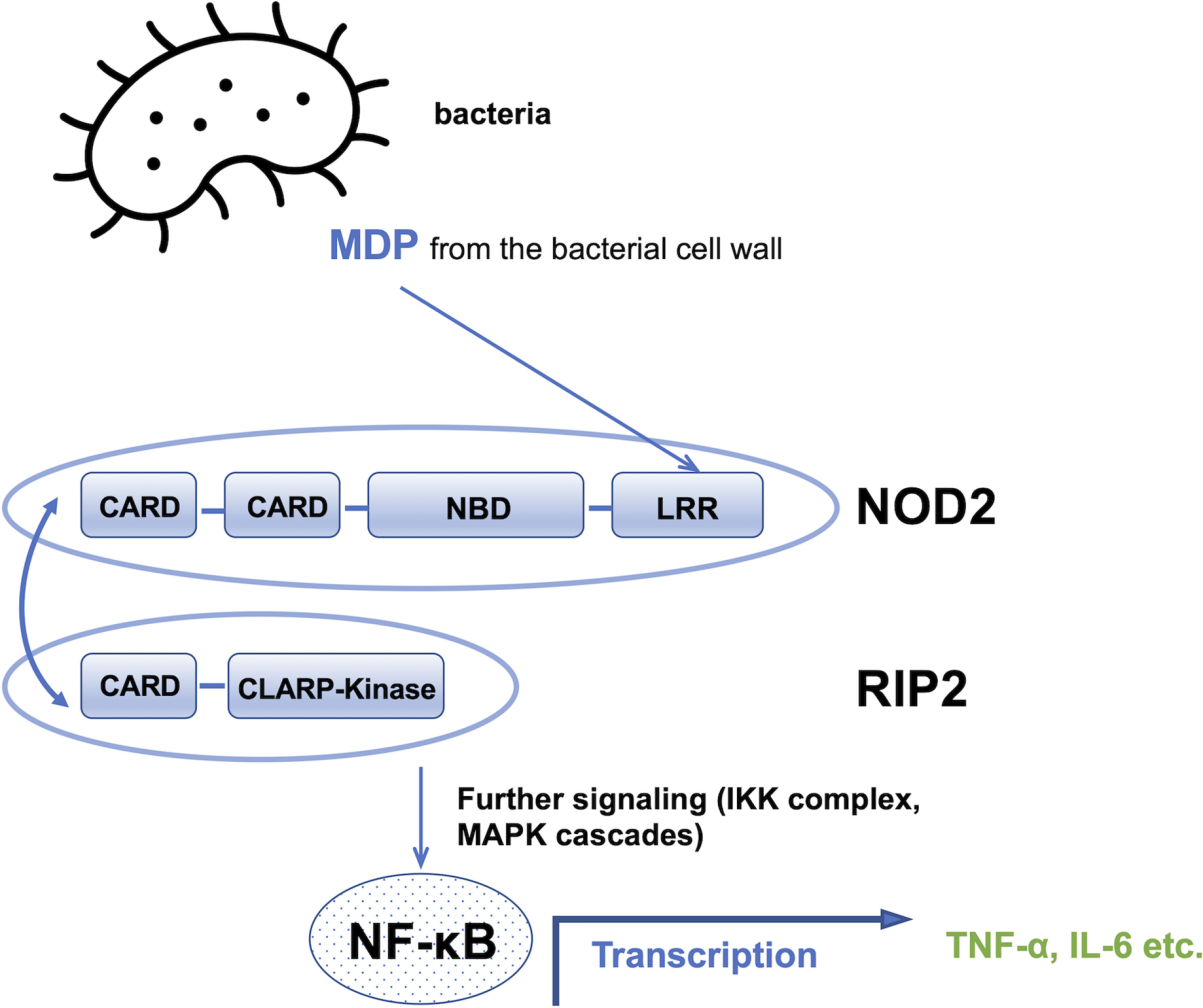
Schematic of the MDP signaling pathway. CARD, caspase recruitment domain; NBD, nucleotide binding domain; LRR, leucine rich repeats domain, NF-kB, nuclear factor kappa-light-chain-enhancer of activated B cells; TNF∝, tumor necrosis factor; IL-6, interleukin 6.

NOD2 belongs to the nucleotide-binding domain leucine-rich repeats family of proteins and consists of three components: the nucleotide binding domain, which is essential for oligomerization and has an ATP binding site within it; the *N*-terminal effector domain with a caspase recruitment domain (CARD); and the leucine-rich repeats domain (7–9). NOD2 is sensitive in terms of MDP recognition which are normally degraded from the bacterial cell wall. Upon recognition of the specific muramyl peptide molecule, it triggers a wide range of inflammatory and antimicrobial responses and stimulates an immune response. These responses include the activation of the nuclear factor kappa-light-chain-enhancer of activated B cells (NF-κB) and mitogen-activated protein kinases (MAPK) signaling pathway (10, 11). When NOD2 detects MDP, it binds to the receptor interacting protein-2 kinase (RIP2) through the caspase activation and recruitment domains (CARD-CARD) interactions, which is necessary for a further signaling step to occur (12). This signaling induces not only NF-κB transcriptional activity through the IKK complex (IkB kinase), but also other cascades involving MAP kinases. These kinases activate the production of cytokines and pro-inflammatory chemokines such as the tumor necrosis factor and interleukin 6, among others, as shown in **Figure 2** (13).

For many years, scientists have been aware of the value of the MDP molecule in terms of its potential use in the treatment of many diseases given its signaling pathway (14, 15). However, many approaches have shown that the MDP molecule exhibits high pyrogenicity and rapid excretion from the body; therefore its structure was modified to exclude several side effects and prolong its presence in the body while not altering its affinity to the NOD2 receptor (16, 17). Certain MDP analogs have already proven to be effective anticancer molecules because of their excellent immunostimulatory activity through the activation of the innate immune system and the induction of genes that trigger inflammatory responses and the production of pro-inflammatory cytokines. Moreover, newly designed and synthesized MDP analogs have also shown antiproliferative and cytotoxic activity in cell models as well as antitumor efficacy in tumor models (18, 19).

## Biological activity of MDP and its analogs: Clinical application

In addition to the antitumor activity described in our review, the activity related to the interaction with the immune system in the context of immunostimulation has found clinical application in the treatment of autoimmune diseases. With the current advances in medicine, the use of antibiotics is becoming more common as infectious diseases spread rapidly. Their extensive use contributes to mutations in the microbial world and the development of the resistance of organisms to antimicrobial drugs. Because mutations of bacteria and viruses occur much faster than the introduction of new vaccines or drugs, it is critical to find new approaches and strategies to effectively combat the same, especially in the context of the growing number of patients with immunodeficiencies. One promising approach to combat drug resistance in organisms is to use activators of innate immunity-otherwise known as immunomodulators-that can work in combination with the etiotropic drugs used in chemotherapy. Such a direction of development in the fight against pathogens results from revolutionary discoveries in the field of immunology and the understanding of the principles of innate immunity (20, 21). Such immunomodulator is MDP, which is considered to be natural immune regulator. Their interaction with intracellular receptors triggers a sequence of processes that ultimately lead to an effective response to foreign or transformed antigens, which can result in both the regulation of the inflammatory response and the development of immune tolerance. The strong innate immune response to MDP and its analogs can be used to develop immunotherapeutic strategies because they can trigger non-specific immunity against viral, bacterial, and parasitic infections (4). This biological activity of MDP allows it to be used in a wide range of medical fields (22).

Although some details of the biological activity of MDP are still unknown, many studies have already been conducted on it, and its association with many important diseases has been discovered. MDP, through activation of the NOD2 pathway, has been implicated, among others, in a number of serious immune dysfunctions as a factor that plays a pathophysiological role in many disorders. This is mainly true for graft-versus-host disease (23) or enhanced mortality during sepsis (24) and the well-known Crohn’s disease (CD) (25). An *in vivo* experiment conducted by Salem et al. (26) also confirmed the antiviral effect of MDP when administered intravenously in mice exposed to influenza A virus (IAV) infection. An effective and commonly used drug in the form of a solution for intramuscular administration is a drug called Polimuramil, which is a muramyl peptide complex. This drug is mainly used in adults simultaneously with antibacterial drugs in the complex therapy of secondary immunodeficiency, i.e. during chronic infectious and inflammatory processes of soft tissues or skin, as well as in the prevention and treatment of surgical infections. The drug works by stimulating the elements of the immune system that are necessary to fight the extracellular forms of bacteria (22).

**Figure 3:**
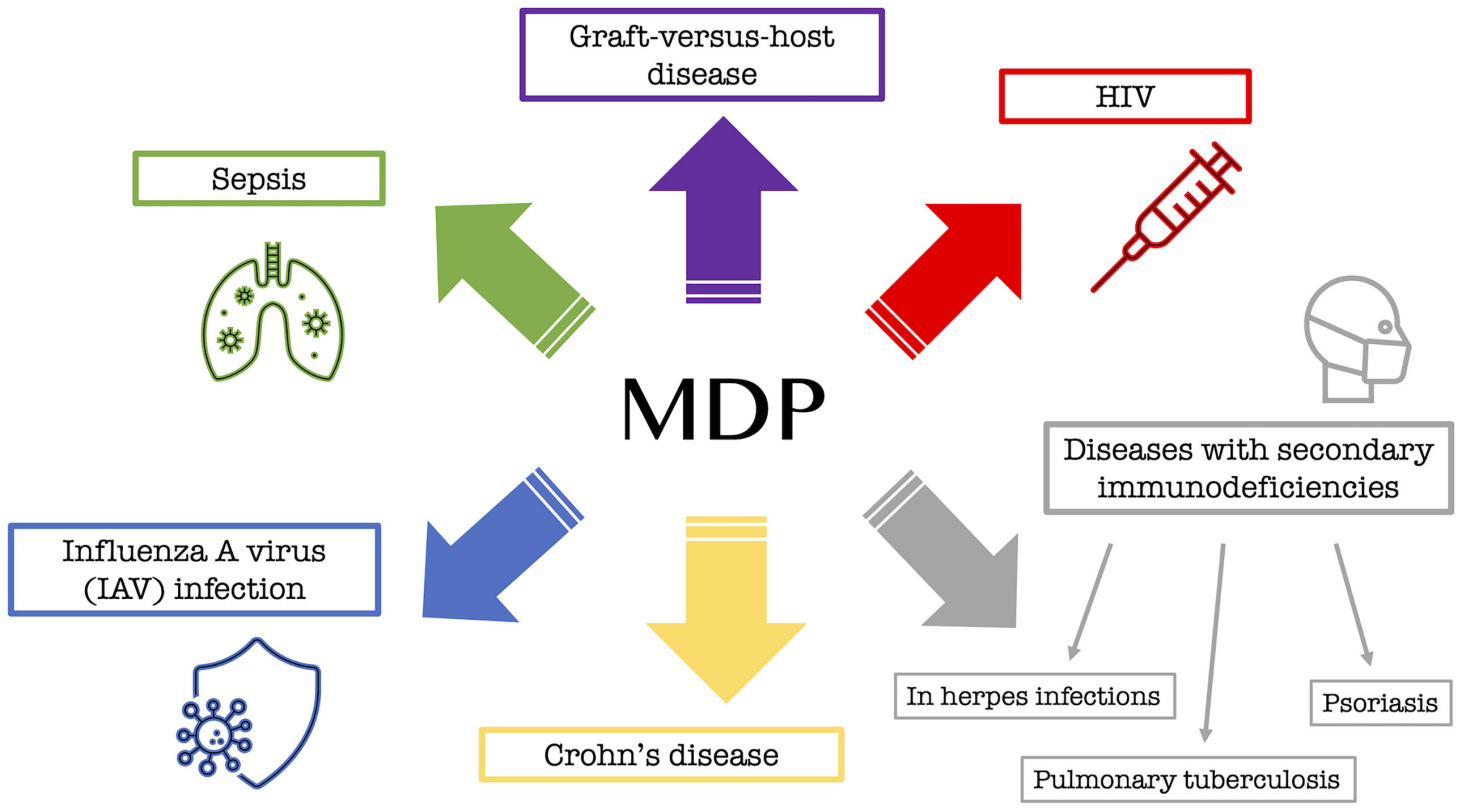
MDP analogs in biological use for the treatment of bacterial or viral diseases.

In addition to MDP itself, a couple of analogs have been discovered that are now being successfully used to fight various pathogens or diseases. Murabutide (N-acetylmuramyl-L-alanyl-D-glutamine-n-butyl ester), an MDP analog, is a safe MDP derivative that enhances host immunity primarily against bacterial infections and exhibits non-specific tumor resistance and the induction of cytokines and chemokines involved in enhancing immune responses (27). Murabutide is administered to HIV-infected patients to regulate multiple pathways, leading to a significant reduction of viral mRNA in their monocytes and dendritic cells, ultimately causing inhibition of viral replication (28). Another substance that is used as a drug, in which the active substance is GMDP (*N*-acetylglucosaminyl-*N*-acetylmuramyl-L-alanyl-D-isoglutamine), has been used since the nineties in the complex therapy of diseases with secondary immunodeficiencies, such as recurrent and chronic infections of the lower and upper respiratory tract at the stage of chronic, acute purulent dermatitis and soft-tissue inflammation (e.g., furunculosis or pyoderma), as well as for herpes infections, pulmonary tuberculosis, or psoriasis (22). **Figure 3** shows the information collected regarding the main biological applications of MDP and its analogs.

## Use of MDP analogs as adjuvants in cancer therapy

**Figure 4:**
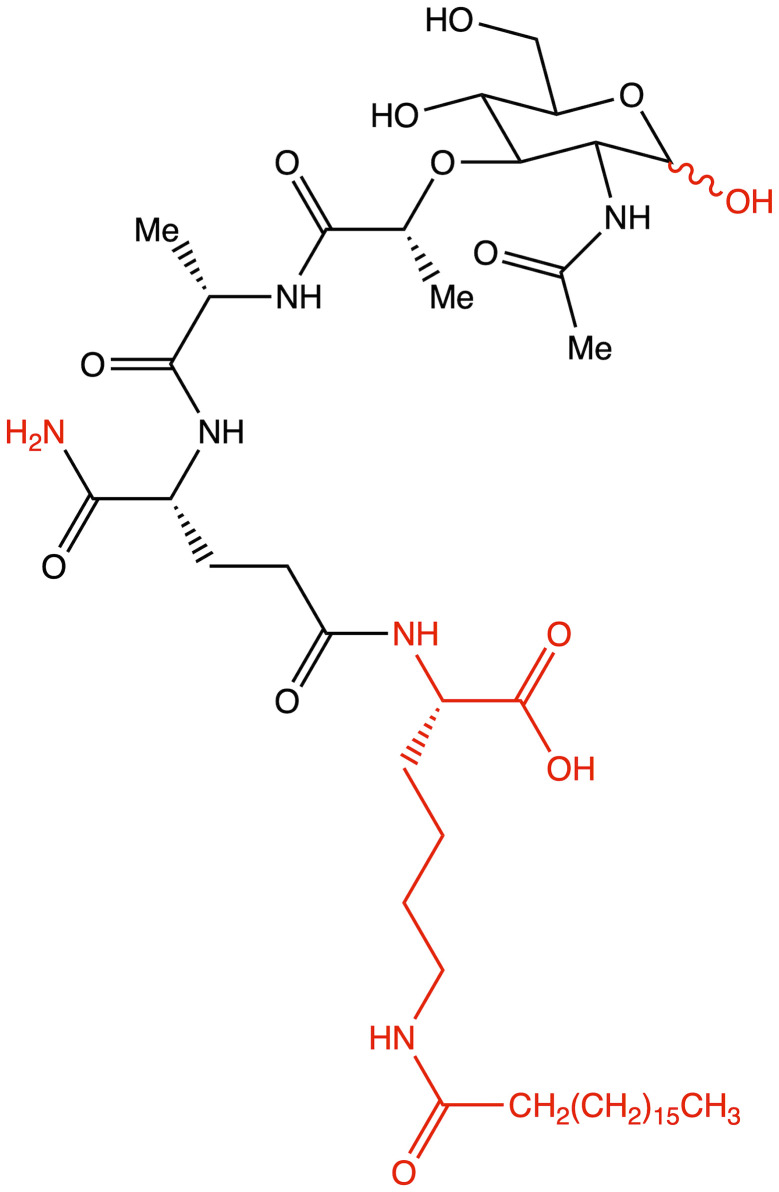
Structure of romurtide with indication of structural differences from the starting MDP molecule (red color).

MDP analogs, due to their beneficial immunostimulatory properties and lack of pyrogenicity, have also been used as adjuvants to cancer vaccines. The adjuvant activity of MDP consists of enhancing the immune response by compounds, which, occurring independently, does not provide a direct stimulatory effect; this situation occurs primarily in the context of vaccines, where MDPs occur next to the antigen (16). MDP is an effective adjuvant, the primary effect of which is to increase the immunogenicity of poorly immunogenic antigens; it also reduces the need for complementary immunization, induces a broader immune response or extends the duration of protection, and reduces the risk of adverse side effects during antitumor treatment (29). Vaccination enables the induction of antigen-specific immune responses and is able to counteract tumors caused by, for example, viruses. In recent years, it has been shown that vaccines can effectively treat established cancers in a significant percentage of patients (**Figure 4**).

The best known adjuvant in medicine, which is also used as an acitvity standard, is Freund’s complete adjuvant (FCA) (30). FCA consists of heat-killed mycobacterial components in an oil emulsion and thus, can induce a strong humoral response as well as a cellular immune response. In 1974, it was discovered that MDP is the minimum structure that is required for the effectiveness of Freund’s complete adjuvant (31). Unfortunately, Freund’s complete adjuvant is highly toxic and causes many messy side effects in humans, making it unfeasible for use in the clinic. MDP, on the other hand, is also too pyrogenic to be used clinically and is rapidly eliminated from the body due to its low molecular weight and water solubility, making it incapable of creating the desired immunotherapeutic effect (30). Therefore, the synthesis of analogs and derivatives of MDP that present similar properties without significant side effects and that would result in a significant improvement in the properties of the parent molecule became an important direction of research (32). The relatively small development in this field may be due to the fact that finding new adjuvants is based on an empirical approach and must meet a number of stringent safety requirements (33). Adverse effects can be tolerated to some extent in cancer treatment but not in the context of adjuvants in prophylactic vaccines, which is why it is so difficult. Despite these difficulties, researchers are taking on the challenge of finding increasingly effective MDP analogs because adjuvant treatment has a significant positive impact on survival and its use is widely accepted (34).

To date, several MDP analogs have been discovered that have reached clinical trials or are being successfully used as adjuvants in vaccines against various diseases (35, 36). However, when it comes to analogs that have shown promising activity in the antitumor context, there are only a few. One adjuvant that has been approved for clinical trials is an MDP derivative (ImmTher) that is a liposomal variation of the drug in the form of N-acetylglucosamine-N-acetylmuramyl-L-alanyl-D-isoglutaminyl-L-alanyl-glycerol dipalmitate. The main indications for its use are osteosarcoma, Ewing’s sarcoma, and colorectal adenocarcinoma with lung and liver metastases. In clinical trials, multiple injections of the drug with concurrent radio- and chemotherapy resulted in neutrophil-induced leukocytosis and a significant increase in tumor-necrosis-factor (TNF) levels (37). Another analog that has been approved for treatment is muramyltripeptide phosphatidylethanolamine (MTP-PE, mifamurtide) embedded in liposomes (named Mepact). It is widely used in adjuvant therapy in many countries for the treatment of osteosarcoma in combination with traditional chemotherapy. Because of the stringent requirements, it is very difficult to find an adjuvant that is non-pyrogenic and assimilates well in the body while having selective cytotoxic effects. Therefore, researchers are intensively searching for new analogs with better activity combined with low pyrogenicity and no side effects.

Knotigová et al. (38) described the study of a series of lipophilic analogs of norAbu-MDP and norAbu-GMDP in the form of liposomes to produce recombinant vaccines for use in the stimulation of innate immunity and of immune cells and tissue regeneration. The present study focuses on the restoration of hemopoiesis in mice that were sublethally and lethally irradiated with γ rays. The ability of the tested analogs to stimulate bone marrow regeneration was demonstrated by determining GM-CFC regeneration 13 days after the sublethal γ-ray irradiation of the mice. The study identified the MT-02 and MT-07 analogs that showed the greatest stimulatory and regenerative abilities after irradiation. Based on the result of the study, it can be said that both derivatives have a strong potential to induce complex regenerative and protective mechanisms, ultimately leading to higher survival rates of lethally irradiated mice. The compounds MT-02 and MT-07 may be promising adjuvants for recombinant vaccines during not only the stimulation of innate immunity but also bone marrow reconstruction after radiotherapy or cancer chemotherapy.

The norMDP compound is currently in phase 1 clinical trials as an adjuvant in the HER-2 vaccine against gastric, breast and ovarian cancer (36). Bekaii-Saab et al. (39) described a study involving the adjuvant norMDP (N-acetyl-nor-muramyl-L-alanyl-D-isoglutamine) as an adjuvant in vaccines containing B-cell epitopes. Constructing such vaccines is a novel method that induces the formation of high-affinity antipeptide antibodies against cancer that help circumvent intrinsic drug resistance. Such vaccines are designed to represent the binding sites of trastuzumab and pertuzumab, which, unfortunately, are often not effective due to patients developing resistance to these drugs. The vaccine combination consisted of two B-cell HER-2 epitopes, namely HER-2 (266–296) and HER-2 (597-626) fused to a measles virus fusion (MVF) protein and ‘promiscuous’ helper T-cell epitope and combined with norMDP. HER-2 is a transmembrane receptor that is overexpressed in many epithelial cancers and associated with more aggressive forms of cancer; therefore, it is a key therapeutic target in many cancers. The results of the study suggested a promising potential benefit of this vaccine over humanized monocolonal antibodies, as no build-up of secondary resistance was observed. The investigational vaccine demonstrated antitumor activity and tentatively indicated that it may circumvent resistance to monoclonal antibody therapy, making it an attractive alternative in the future.

The analog described as a potential and effective adjuvant is GMDP-A, which is a derivative of GMDP. It has shown high efficacy in trials as a component of complex anticancer therapy. It is now ready for clinical trials as an integral part of the treatment of transplantable cancers, including B16 melanoma and P388 lymphocytic leukemia. Through preliminary studies, it has been found to inhibit tumor growth in more than 95% of cases, reduces the incidence of metastasis, and results in longer animal lifespans and a 30% reduction in the use of the cytostatic drug cisplatin (Cis) [40].

Guzelj et al. (40) conducted a study focusing on desmuramylpeptide analogs. They discovered an MDP analog, compound 75, which increased the cytotoxic activity of peripheral blood mononuclear cells against malignant K562 and MEC1 cancer cell lines. Additionally, it induced an increase in dendritic cell-mediated T-cell activation and exhibited immunostimulatory effects on peripheral blood mononuclear cells at the transcriptional and protein levels. The compound, as an adjuvant used in liposomes, exhibited greater adjuvant activity than the starting MDP molecule when tested with mice. The researchers reported that, due to the lipophilic tail in the structure at the C18 molecule, the compound exhibits such good and desirable activity and is, therefore, a promising analog that can be tested in clinical trials as a potential adjuvant with anticancer activity.

**Table 1** summarizes the discussed MDP analogs with adjuvant properties in anticancer therapies described in the last 10 years along with the structures and results of the studies performed.

**Table 1:** MDP analogues for potential adjuvant use in anticancer therapy.

	CHEMICAL STRUCTURE	BIOLOGICAL ACTIVITY	REF.
**1.**	**MT02** 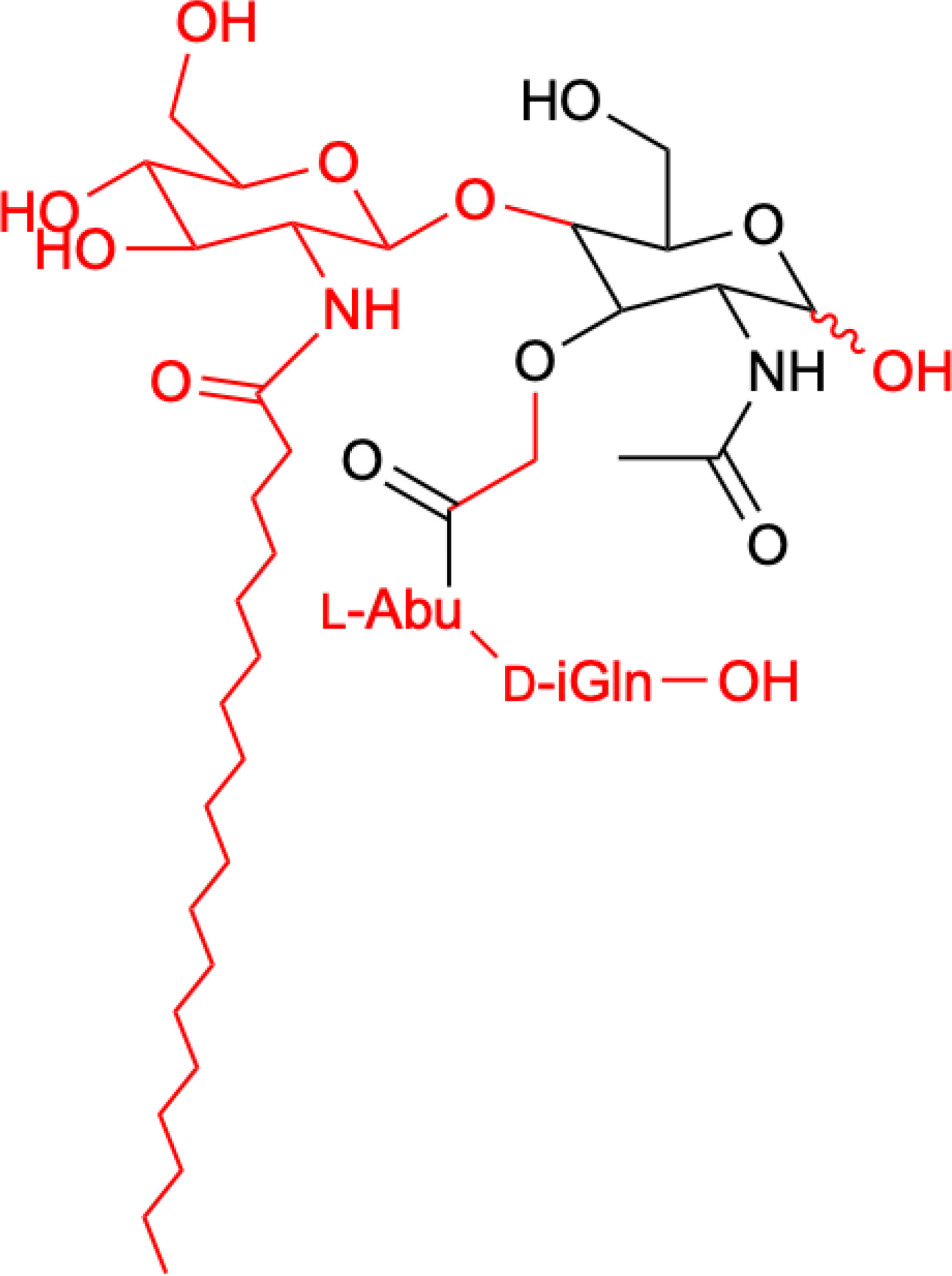	**Both derivatives exhibit significantly reduced pyrogenicity compared to MDP and have no side effects when used in animals (ICR mice, female, age of 3 months). They show potential antitumor applications due to their strong bone marrow restorative properties, potentially for use after radiotherapy or chemotherapy of cancer.**	(38)
**2.**	**MT07** 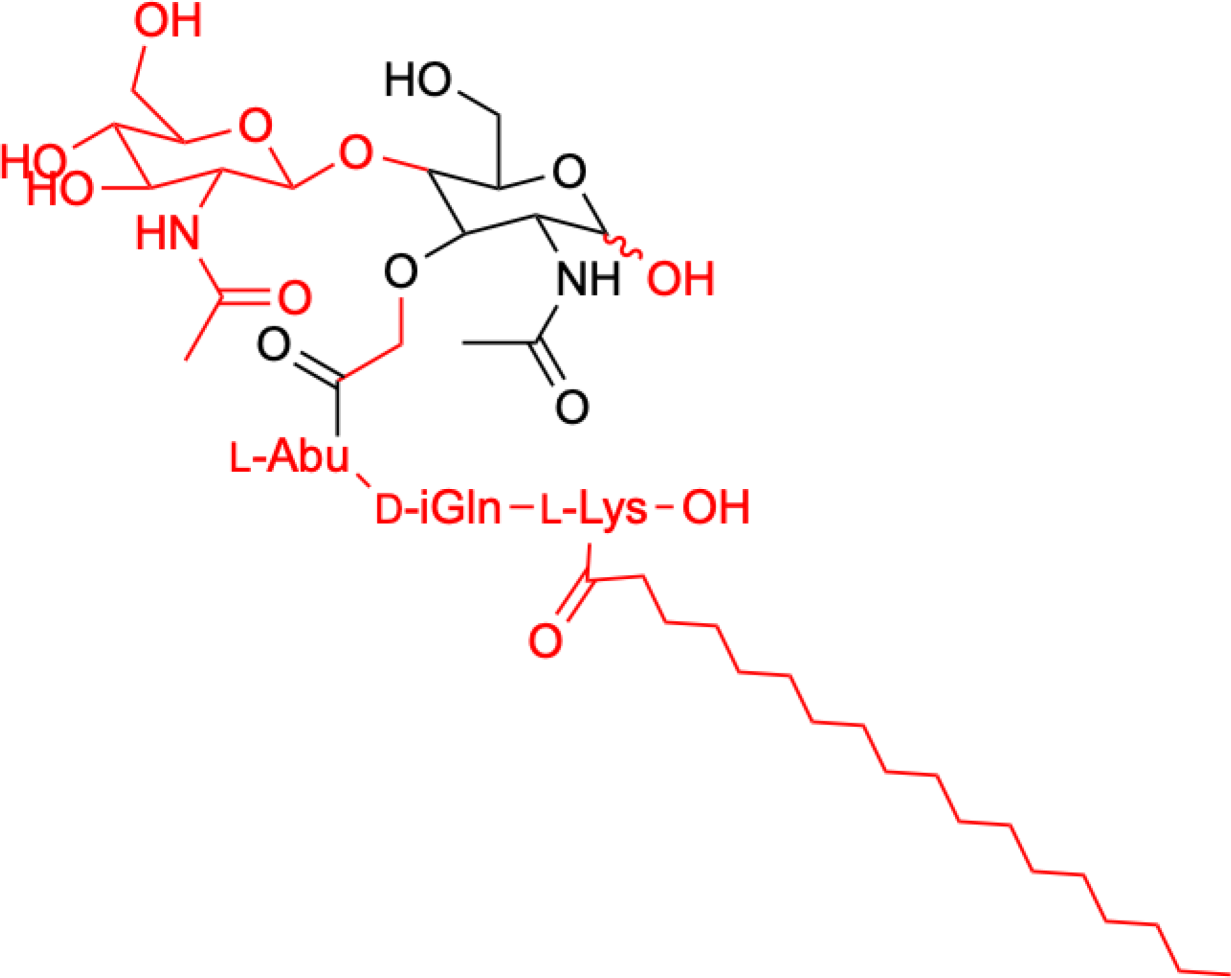		
**3.**	**nor-MDP** 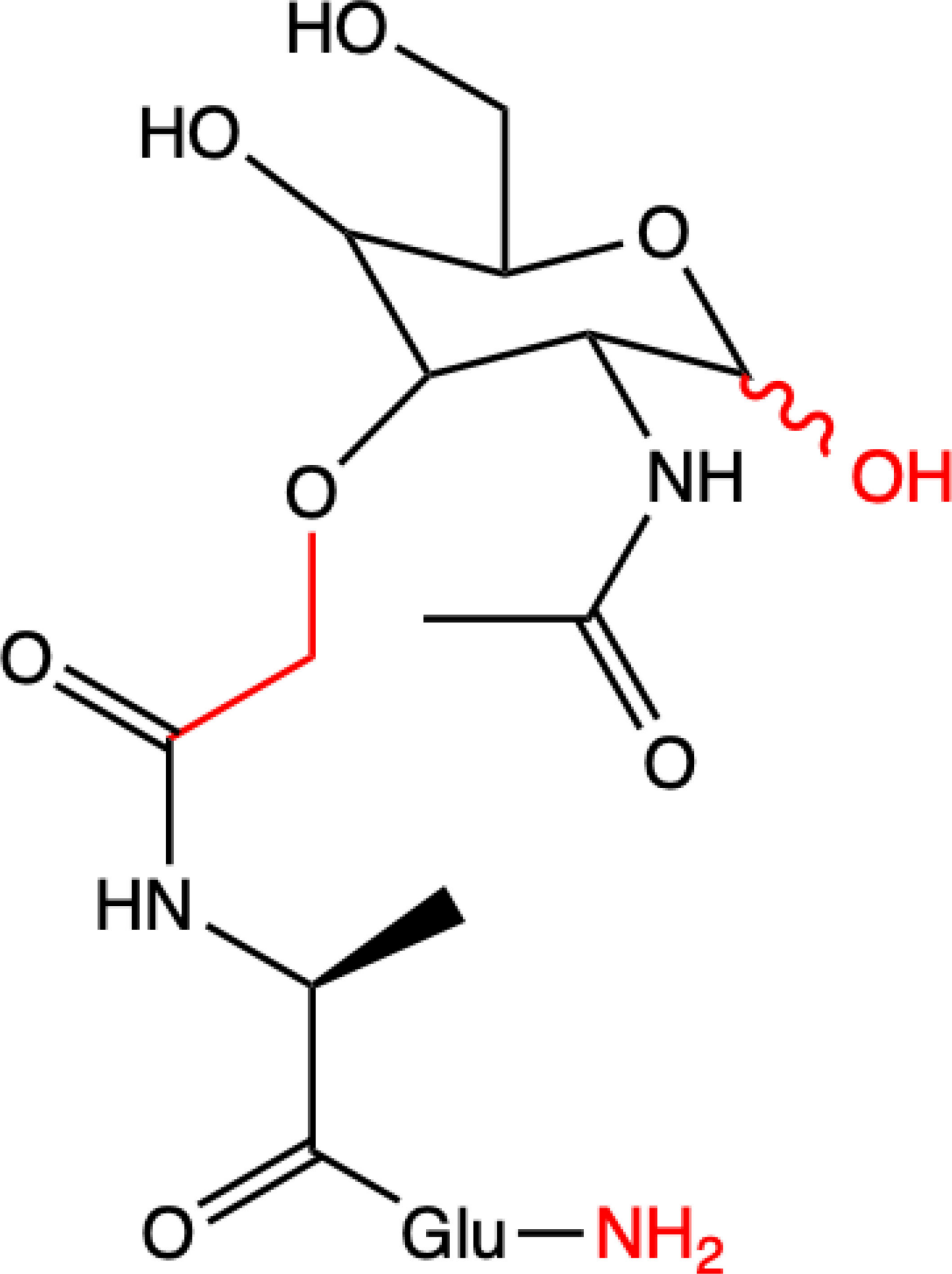	**Adjuvant in a peptide cancer vaccine that produced a sustained humoral response by inducing antibodies recognizing the HER-2 receptor in most patients. Most of the patients' antibodies that were produced in response to the vaccine showed potent antitumor activity and defense mechanisms (induction of ADCC and apoptosis, inhibition of proliferation and phosphorylation).** **The human breast tumor cell line BT-474**	(39)
**4.**	**GMDP-A** 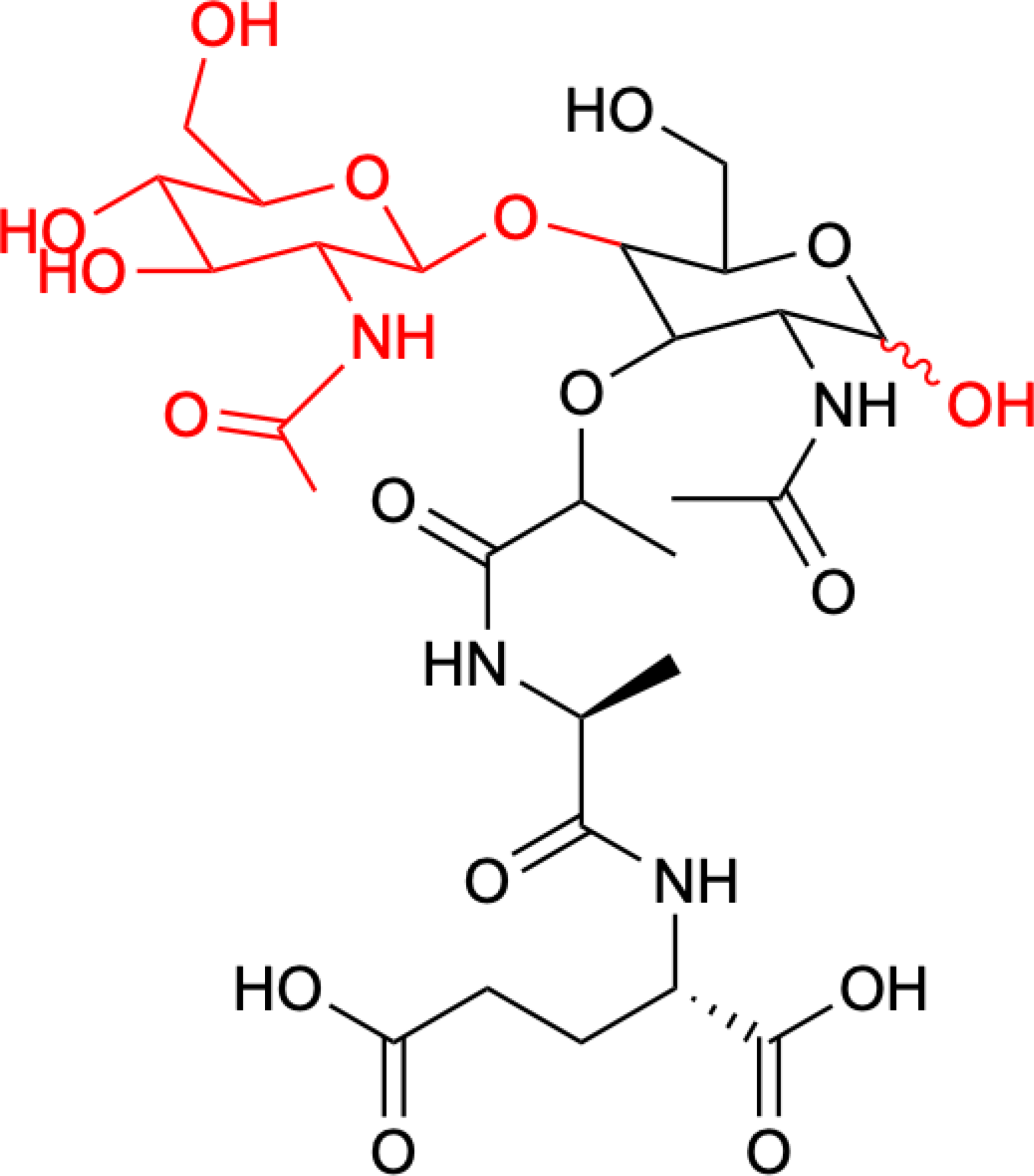	**Analogues inhibits tumor growth in more than 95% of cases, reduces the incidence of metastasis** ***In vivo*, B16 melanoma and P388 lymphocytic leukemia**	(41)
**5.**	**Compound 75** 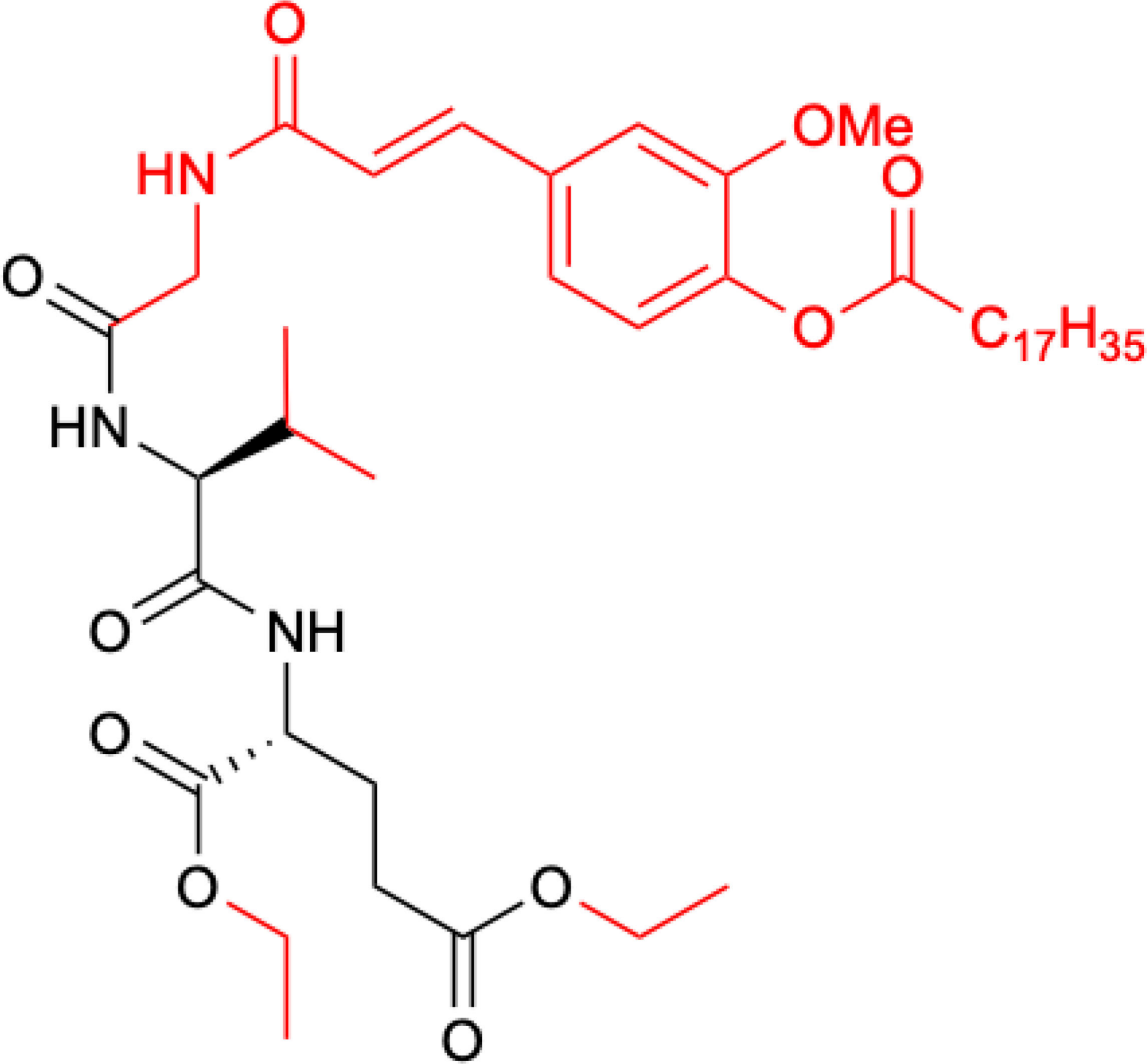	**The compound showed a significant increase in cytotoxicity of PBMCs against both human cancer cell lines: lymphoblasts** **K562: chronic myelogenous leukemia cell lines and MEC1: B-chronic lymphocytic leukemia cell line** **The compound also had better pharmacokinetic properties than MDP - better adjuvant properties and lower pyrogenicity.** ***In vitro*: HEK-Blue NOD2 cells from human embryonic kidney cells HEK293** ***In vivo*: NIH/OlaHsd mice**	(40)

(**green**, research model/cell lines; **red**, changes in structure relative to the MDP).

## Potential anticancer activity of MDP analogs: Cellular and animalmodel studies

Anticancer activity and the establishment of new cancer treatments is of paramount importance to current medicine and is one of the most developing and thriving fields. The broad spectrum of biological activity of PGN metabolites has meant that their therapeutic use in particular has been heavily researched since they were discovered. However, unfortunately, the MDP molecule has several properties that cause problems and make it very difficult - if not completely impossible - to use directly in clinical applications.

MDP is a hydrophilic molecule that is very easily excreted from the body once it has been administered. More than 50% of it is removed by the kidneys within 30 minutes after administration in mice, while more than 90% is eliminated within 2 hours (42). The molecule in question may also undergo hydrolysis in the host body during circulation. In studies involving rat serum, it was discovered that MDP degrades to its monomeric components, although this process is not as rapid as the excretion of the molecule. The broad modulatory effects of these molecules on the immune system can also be a major problem, resulting in unwanted effects. It has been shown that MDP can be both pyrogenic, which has hindered its use in immunotherapy (43, 44) and somnogenic (45). To overcome these adversities, intensive efforts have been made to develop variants and modify the MDP molecule to obtain not only better pharmacological properties and increased potency but also higher bioavailability and limited side effects *in vivo* (42).

Ibrahim et al. (46) investigated the antitumor and immunomodulatory effects of co-treatment based on MDP with or without Cis and bovine lactoferrin (bLF) in mice with Ehrlich solid tumor (ESTs). It was concluded that the combination of MDP and bLF could stimulate an innate but also an adaptive immune response, and this could lead to an optimal therapeutic immune response in an Ehrlich carcinoma murine model. The simultaneous treatment of EST mice with bLF and MDP significantly reduced tumor size compared to untreated tumor-bearing mice, indicating antitumor effects.

Research efforts have also largely focused on structural modifications and the identification of the essential elements of structure and structural flexibility associated with the three monomeric MDP residues (47). Structural modification studies have led to the synthesis of more stable MDP derivatives and the selection of active compounds with a low toxicological profile, leading to their approval for further preclinical and clinical studies (48). Unfortunately, there are still relatively few analogs that have demonstrated anticancer properties and have been approved for the treatment of cancer patients. Hence, in this review, we focus on the current status of the structure-activity relationship and the anticancer potential of MDP and its analogs.

To date, two MDP analogs have been approved to treat patients: mifamurtide and romurtide. An MDP analog that has been discovered and successfully introduced for treatment is mifamurtide. Mifamurtide is a potent stimulator of innate immunity, a non-specific immunomodulator, and a synthetic analog of a bacterial cell wall component (49). Through its presence in phospholipid vesicles, there is the possibility of easy uptake by tissue macrophages, which makes activated macrophages directly attack and kill cancer cells and prolongs its presence in the lungs. Mifamurtide increases the production of adhesion molecules on the surface of macrophages, which promotes action on cancer cells. In addition, cytokine production promotes secondary activation of other immune effectors such as T-lymphocytes, granulocytes, NK cells, and dendritic cells. Mifamurtide is indicated for use in young adults, especially children and adolescents, for the treatment of osteosarcoma (50). The mechanism of action of mifamurtide may increase overall survival (OS) and event-free survival (EFS), as a significant proportion of metastases in osteosarcoma patients occur in the lungs (49).

Biteau et al. (18) demonstrated that L-mifamurtide (L-MTP-PE) inhibits the dissemination and development of lung metastases and also showed the slowing of tumor growth in mice. They described that mifamurtide has a unique mode of action in that, by modulating the immune system, it indirectly affects cytotoxicity rather than directly affecting cancer cells. The researchers also showed that L-MTP-PE alone had no effect on osteosarcoma cell proliferation: MOS-J and KHOS *in vitro*; but the combination of L-MTP-PE with zoledronic acid significantly inhibited the progression of primary osteosarcoma. This study is the first to report the strong tendency of L-MTP-PE to inhibit the spread of lung metastases in mouse models of osteosarcoma - both syngeneic (MOS-J) and xenogeneic (KHOS) preclinical mice models of osteosarcoma. Human osteosarcoma cell line KHOS and mouse osteosarcoma cell line K7M2 as well as a syngeneic (MOS-J) and xenogeneic (KHOS) mouse models of osteosarcoma were used in the described studies.

Moreover, chemotherapy with mifamurtide has been shown to improve lifetime efficacy compared with chemotherapy alone for both non-metastatic and metastatic osteosarcoma. The relative efficacy of the therapy was higher in metastatic osteosarcoma than non-metastatic osteosarcoma, while the absolute efficacy was higher in non-metastatic osteosarcoma than in metastatic osteosarcoma (51). The results of a phase II clinical trial using L-MTP-PE alone demonstrated an apparent increase in both disease-free survival and long-term survival of patients with recurrent osteosarcoma (52). In contrast, the results from a phase III clinical trial, where mifamurtide was used in combination with chemotherapy, showed improved six-year survival (53, 54).

Further, Taçyıldız et al. (55) demonstrated in their study that mifamurtide is an effective adjunct to chemotherapy and improves treatment outcomes in newly diagnosed cases of osteosarcoma without metastatic disease. Based on current scientific knowledge, approximately 80-90% of newly diagnosed osteosarcoma patients without detectable metastatic disease have micrometastatic disease. These are often subclinical or undetectable using the current diagnostic methods (56); hence, it is extremely important to use a chemotherapy adjuvant that would act against metastasis and delay its occurrence. In the described studies, mifamurtide delayed the median time to metastatic disease from 4 months to 7.2 months in patients with positive surgical margins compared to patients who received chemotherapy without the addition of mifamurtide (55). There was also a delay in the development of distant metastases from a median of 5 months to 20 months in patients who had distant metastases as a first event. Here, again, the time from the diagnosis of the disease was compared between the groups receiving and not receiving mifamurtide. Furthermore, for local recurrence, mifamurtide reduced the incidence of metastatic disease. The structure of mifamurtide is shown in **Table 1**.

The second analog that is used to treat patients is romurtide. This is a compound that is made by attaching ε-N-stearoyl-L-Lys to the MDP scaffold (57). Romurtide stimulates macrophages to release cytokines as well as colony-stimulating factors, which can increase platelet and white blood cell counts (58, 59). It is currently used primarily to treat leukopenia after chemotherapy. Studies have also proven that intradermal injection of IFNb and various concentrations of romurtide lead to a reduction in primary tumor size and improved lymphocytic infiltration using a B16-F10 subcutaneous melanoma model in mice (19). A study of patients with gastrointestinal cancer also found an increase in the number of cases with increased lymphocyte counts when using romurtide compared to a group that did not use it. These results may indicate that romurtide has a restorative effect on thrombocytopenia that is similar to that of leukocytopenia when given as concomitant therapy with anticancer drugs and/or radiation in patients undergoing intensive treatment for gastrointestinal cancer (60). It is also a very promising compound for cancer patients who receive high doses of carboplatin, which can induce thrombocytopenia due to severe myelosuppression. Namba et al. (61) demonstrated that romurtide accelerated platelet regeneration, thereby shortening the duration of monkey thrombocytopenia.

Pahl et al. (62) described a study in which the addition of IFN-γ to L-MTP-PE was found to improve survival in mice with murine renal adenocarcinoma and inhibit metastasis (63). They also reported that the clinical efficacy of L-MTP-PE for treating patients with osteosarcoma may be enhanced by attaching a macrophage-priming signal such as IFN-γ. Buteyn et al. (64) investigated L-MTP-PE in combination with IFN-γ in a disease context other than osteosarcoma, namely in relation to the use of the drug in human acute myeloid leukemia (AML) cells. An *in vivo* study showed that activation of NOD2 by L-MTP-PE + IFN-γ resulted in the direct induction of caspase-induced apoptosis and the release of pro-inflammatory cytokines in AML blasts. Unfortunately, L-MTP-PE alone does not induce an inflammatory response; when combined with IFN-γ, it leads to strong activation. It has also been noted that patients with an immature natural killer (NK) cell profile have reduced overall survival as well as extremely reduced three-year relapse-free survival (65). Importantly, NK cell maturation can be stimulated by a combination of L-MTP-PE and IFN-γ and is, therefore, considered to have very high therapeutic potential and may merit further investigation (64).

Another MDP analog – MTC-220 conjugated with paclitaxel (PTX) - has been discovered that demonstrates antitumor and antiproliferative properties (66). Paclitaxel has clinical applications and is used in cancer chemotherapy (67), but it has been associated with several serious problems. Such problems include drug resistance, toxicity, and poor solubility (68). It was also recently discovered that it can contribute to metastasis (69, 70), which has limited its use for fighting against metastasis.

Ma et al. (71) described the effect of MTC-220 on proliferation and metastasis in xenograft models using human breast (MDA-MB-231, MCF-7), ovarian (A2780, ES-2), and lung (H460, A549, H1975) tumor cell lines. The experimental data indicate that this analog effectively inhibited the growth of several types of tumors *in vivo*, notably human breast and lung cancer tumors. MTC-220 also inhibits the growth and metastasis of transplanted cells and have antimetastatic effects on Lewis lung carcinoma (LLC), 4T1 breast cancer cells, and in the highly invasive and metastatic 4T1 mammary carcinoma model *in vivo* - apparently inhibiting tumor growth and lung metastasis while also leading contributing to significantly fewer metastatic nodules in the lungs of mice compared to controls. Moreover, MTC-220 decreases the accumulation of myeloid-derived suppressor cells (MDSCs) in the spleen and bone marrow of 4T1 tumor-bearing mice. Mechanistic studies have also shown that this analog can inhibit the mRNA levels of inflammatory cytokines, such as TNF-α, in tumor tissue.

Dong et al. (72) described a molecular strategy to sensitize the response to chemotherapy by antagonizing NOD2 inflammatory signaling. They suggested that an NOD2 antagonist might be a potential agent that could help in treating non-small-cell lung cancer (NSCLC) and synthesized multiple derivatives of MDP, specifically MTC-220. The most promising non-degradable derivative, DY-16-43, was characterized as an NOD2 antagonist in an experiment involving the HEK-Blue hNOD2 cell line with secreted alkaline phosphatase (SEAP). The compound has been shown to increase sensitivity to PTX therapy and largely prevent tumor metastasis. By altering the structure and replacing muramic acid with cinnamic acid derivatives, the biological function of the molecule as an antagonist was reversed, and this made it a better option for cancer treatment than therapy with PTX alone. PTX consists of two main fragments: 10-deacetylbaccation III (10-DAB) and a side chain. To test which of these two fragments contributes the least to antagonizing NOD2, other conjugates were also synthesized. This experiment showed that the full structure of PTX is necessary to maintain the antagonistic activity through MDPs activating NOD2 signaling, as the compounds labelled 24-28 and lacking the paclitaxel structure showed significantly lower inhibitory percentages at the concentrations tested [73]. During compound synthesis, various cinnamic acid derivatives were attached to the MDP pharmacophore, which is the *N*-terminus of the dipeptide (L-Ala-D-isoGln). Of these, the compounds labelled 22 and 23 presented the highest level of antagonistic activity, and 22 was chosen as the best molecule because it showed better solubility under the test conditions. The study demonstrated that compound DY-16-43 is capable of significantly enhancing antitumor efficacy in mice with LLC tumors, demonstrating that it could be a potential adjuvant therapy for the treatment of NSCLC.

Wen et al. (73) described a new compound called salutaxel, which is a conjugate of docetaxel and an MDP analog. Docetaxel is recognized as a very active chemotherapeutic agent against a variety of cancers, so studies involving it could be promising. Suggesting that properly designed conjugate prodrugs exhibit properties that initiate multiple signaling pathways that lead to antitumor activities and often have superior physicochemical properties or pharmacodynamic/pharmacokinetic profiles, the authors synthesized a pair of MDP-derived conjugates. The syntheses and antitumor studies of multiple conjugates of docetaxel and MDP analogs revealed a lead compound, MDC-405 (designated 2), that showed an excellent pharmacological profile against 4T1 breast tumor growth in mice and against metastasis. However, it was not an ideal candidate due to its unsatisfactory physicochemical properties - most notably, its extremely poor water solubility. To improve its water solubility while retaining its positive anticancer properties, another compound with a carboxylic acid at the C-terminus was synthesized, which is the described salutaxel. This, in turn, demonstrates high efficacy together with satisfactory pharmacological properties. Finally, the compound inhibits the growth of several types of cancer *in vivo*, particularly human breast (MDA-MB-231) and lung (H1975) tumors, more effectively. It also exhibits better properties than docetaxel in terms of its ability to prevent tumor growth and metastasis, making it a promising anticancer drug candidate.

Xie et al. (74) developed a novel drug delivery system that harnesses the ability of innate immune cells to act on cancer cells and uses macrophages as nanoparticle carriers and navigators to specifically deliver drugs to tumors. They used a derivative of MDP, muramyl tripeptide (MTP), as an enhancer of the nanoparticle-loading efficiency. For the study, THP-1 cells were used as the nanoparticle carrier; this is a human leukemia cell line that has an affinity to cancer cells and other inflammatory cells (75). To maximize the antitumor effect and minimize adverse effects on leukocytes, a substance called vemurafenib, which is a drug specifically used for the treatment of melanoma in nanoparticles, and the described MTP, - which is used to increase the efficiency of nanoparticle uptake by THP-1 cells, - were selected as the therapeutic agents, resulting in a complex named MTP-BPLP-PLA-PLX4032 (74). Following *in vitro* studies, the active binding of THP-1 cells to melanoma cells was confirmed in the presence of the nanoparticles, which were delivered to the melanoma cells and released a drug that killed the cancer cells. The studies described here indicate that the MDP derivative plays an important role in the use of nanoparticles in anticancer research, which may be a clue for the development of other immune cell-based systems for targeting cancer cell treatment.

Samsel et al. (14) synthesized a series of adenosine-conjugated MDP(D,D) and norMDP(D,D) derivative conjugates that could act as immunosuppressive agents. The cytotoxicity of these compounds was evaluated against lymphoid cell lines and activated peripheral blood mononuclear cells (PBMCs) as an *in vitro* model of immunosuppression. Two leukemia models were used: mouse lymphocytic leukemia L1210 and the human cell line Jurkat, which is also based on T-cells. Although most of the synthesized derivatives had no effect on cancer cell viability, two compounds were identified that had this characteristic: 8f and 8g. They did not yield significantly higher cytotoxicity against leukemia cells compared to MDP and norMDP, they were significantly less toxic against non-cancerous cells, which qualifies them for further examination in future *in vivo* studies. These derivatives could potentially replace currently used drugs, thus minimizing side effects and reducing toxicity for patients.

**Table 2** displays all the discussed MDP analogs with anticancer properties and with cytotoxic properties *in vitro* that have been described in the last 10 years along with the structures and highlights of the studies conducted.

**Table 2:** MDP derivatives with cytotoxic /antitumor activity.

	CHEMICAL STRUCTURE	BIOLOGICAL ACTIVITY	REF.
**1.**	**L-mifamurtide** 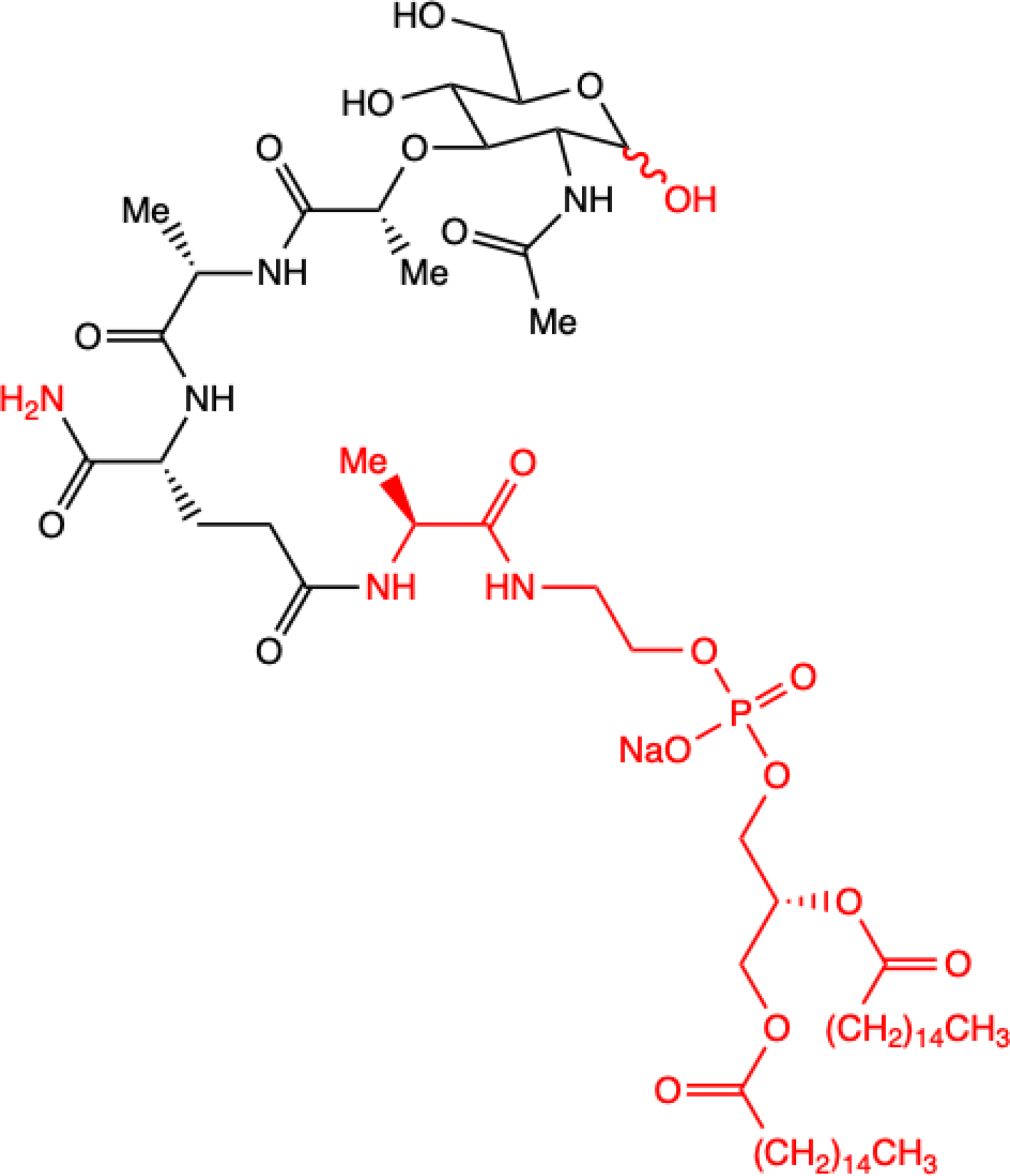 **Conjugation of MDP-L-alanine to the phospholipid dipalmitoyl phosphatidylethanolamine**	**Inhibition of lung metastasis dissemination and development, slowing tumor growth in mice model.** **Alone has no effect on osteosarcoma cells proliferation: MOS-J and KHOS *in vitro*.** **With Zoledronic Acid combination significantly inhibited primary osteosarcoma progression.** **Human KHOS and murine K7M2 osteosarcoma cell lines; syngeneic (MOS-J) and xenogeneic (KHOS) mice model of osteosarcoma**	(18)
**2.**	**MTC-220** 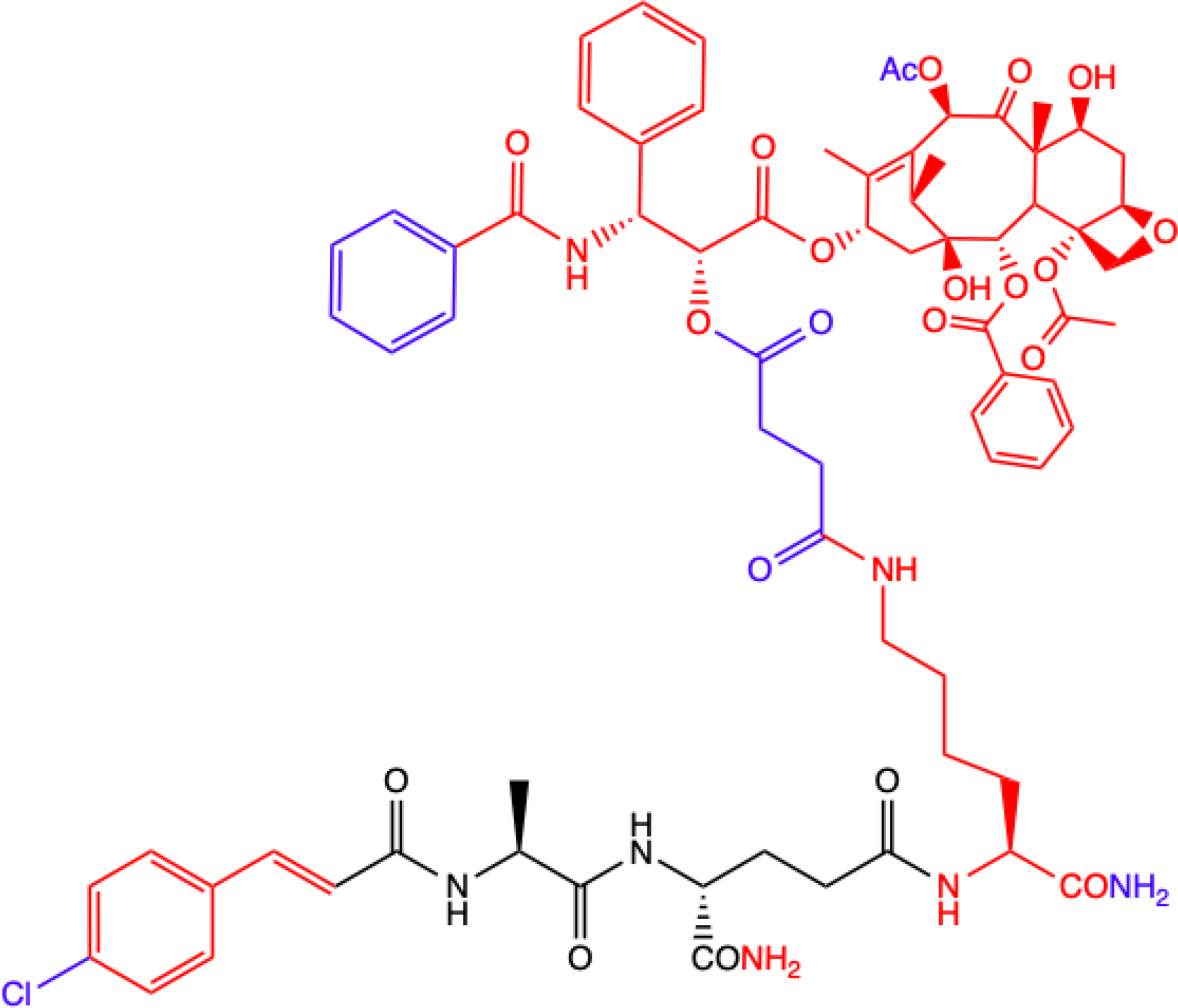 **Full structure of paclitaxel (PTX) combined with MDP analogue – MDA; 4-Cl substituted ring**	**Inhibition of tumor growth and metastasis in transplanted Lewis lung carcinoma (LLC) cells and breast cancer 4T1 cells mouse model.** **Suppressed Myeloid Derived Suppressor Cells (MDSCs) accumulation in the spleen and bone marrow.** **Suppresses inflammatory cytokines in tumor tissue.** **Human breast (MDA-MB-231, MCF-7), ovarian (A2780, ES-2), and lung (H460, A549, H1975) tumor cell lines; 4T1 mammary carcinoma; *in vitro* and in C57BL/6 mice**	(71)
**3.**	**DY-16-43** 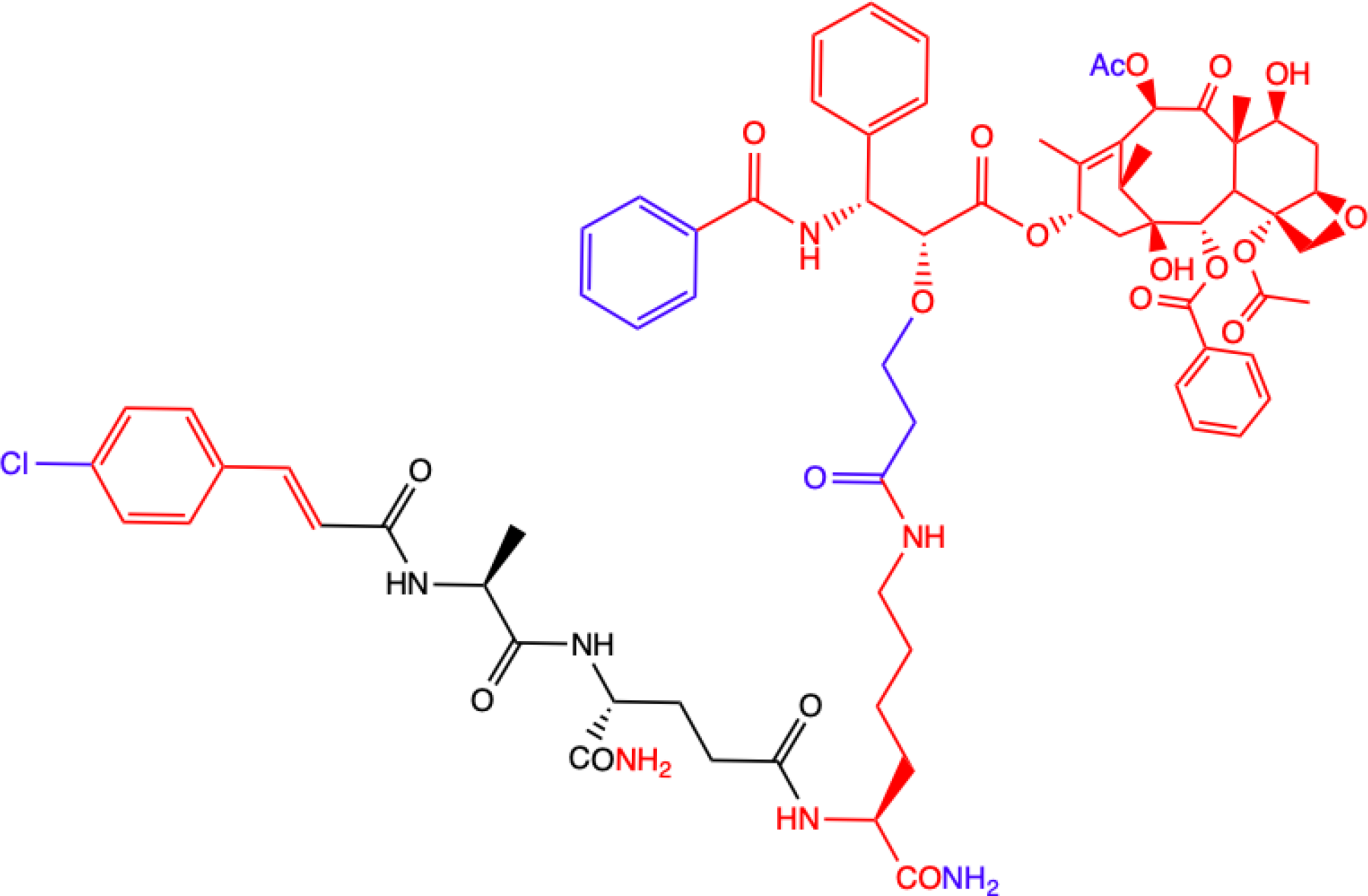 **Full structure of PTX combined with; 4-Cl substituted ring**	**Sensitizes to PTX therapy and prevented metastasis in LLC mouse model – represents a potential adjunctive therapy for the treatment of NSCLC.** **HEK-Blue hNOD2-secreted alkaline phosphatase (SEAP) reporter cell line; human peripheral blood mononuclear cell (PBMC)-derived macrophages; Lewis lung carcinoma (LLC) tumor-bearing mice**	(72)
**4.**	**Compound nr 23** 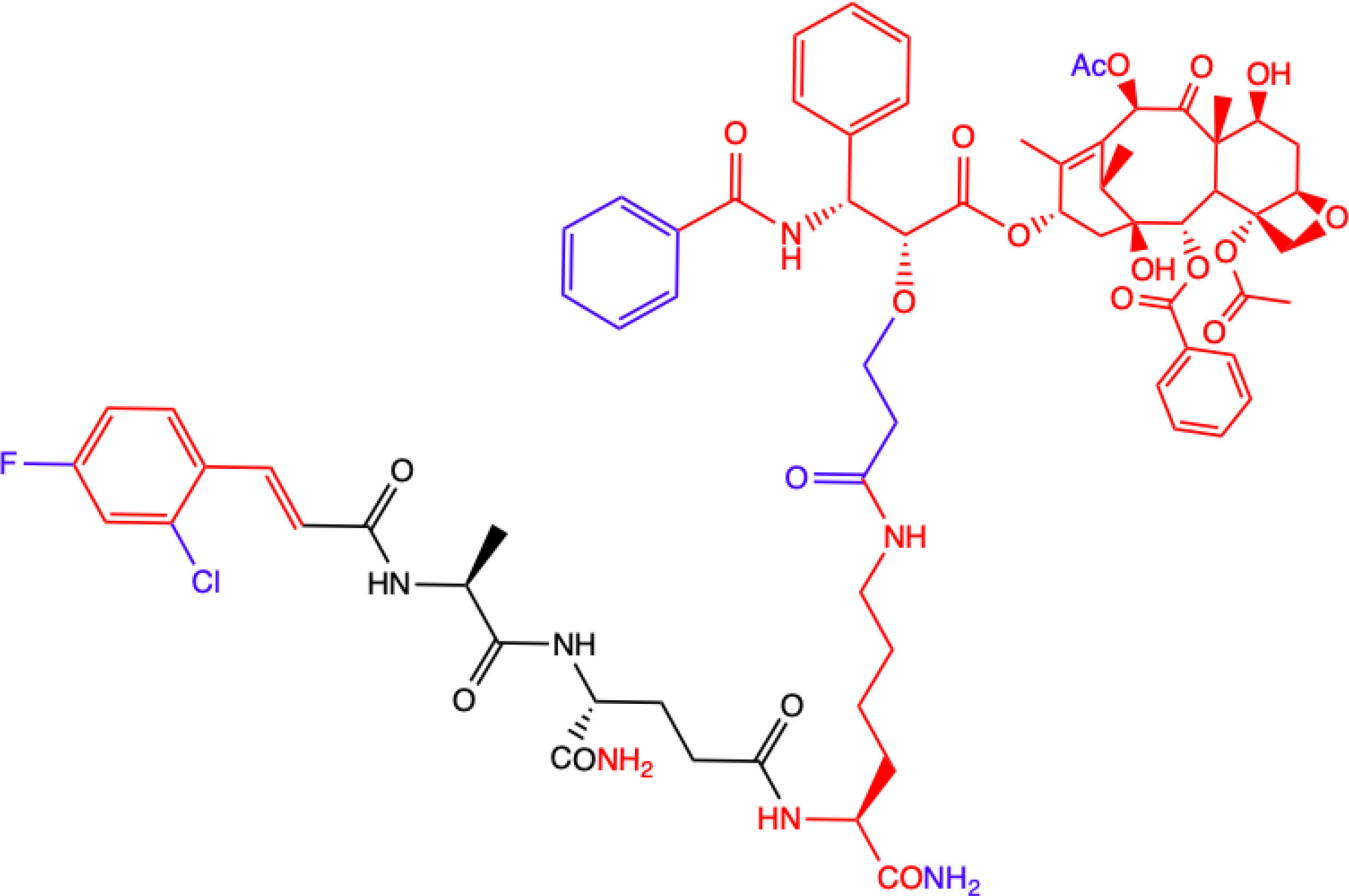 **Full structure of paclitaxel (PTX) combined with MDA**	**This compound, like DY-16-43 showed the highest level of antagonistic activity via MDP activating NOD2 signaling but has poorer solubility under the conditions tested.** **HEK-Blue hNOD2 cells; Lewis lung carcinoma (LLC) tumor-bearing mice.**	
**5.**	**Compound nr 24** 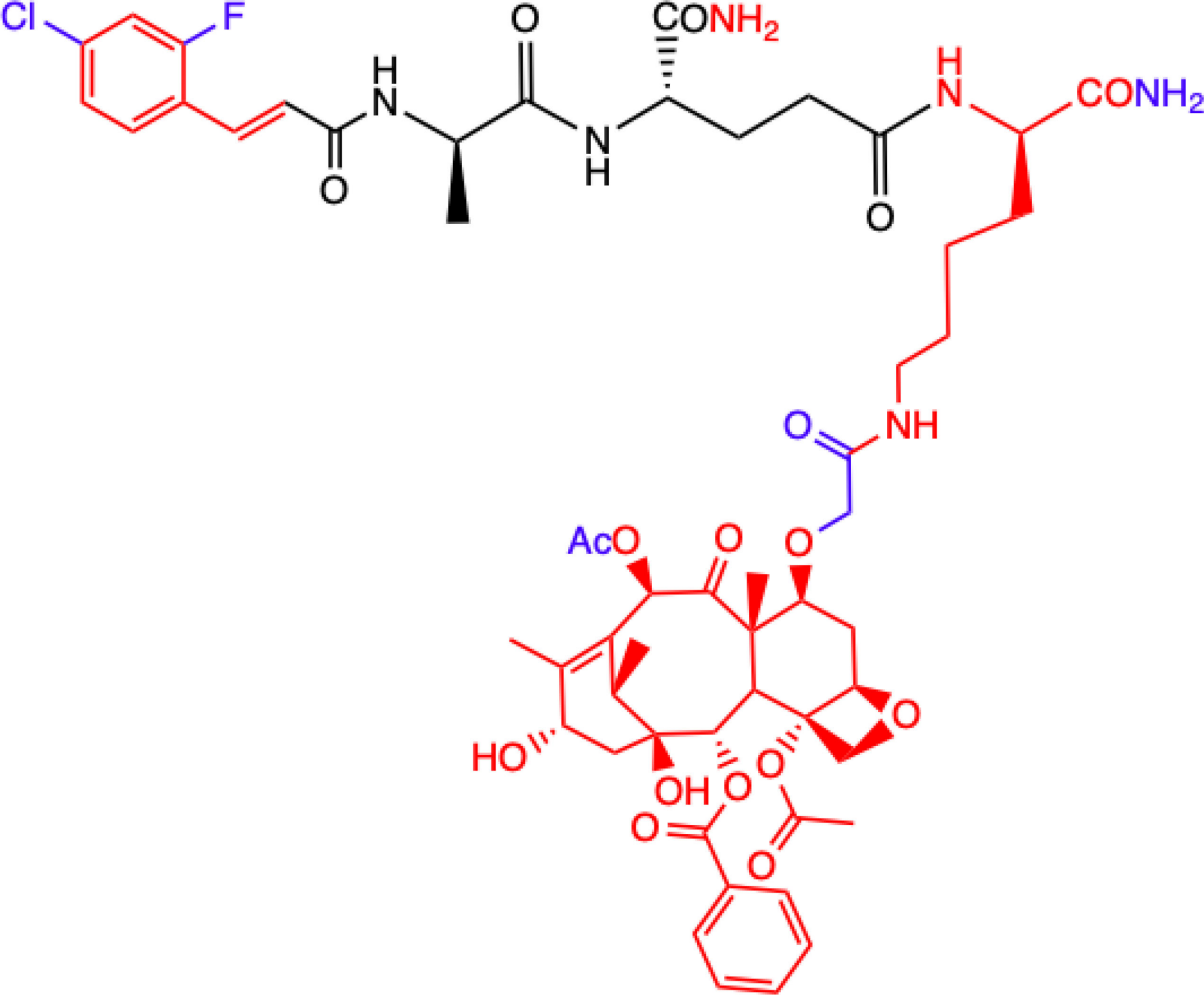 **7-OH substituted 10-OAc-10-DAB, without side chain of PTX; MDA - 4-Cl and 2-F substituted ring**	**The experiment showed that the full structure of PTX is necessary to maintain antagonistic activity via MDP activating NOD2 signaling, which results in sensitization of a response to chemotherapy in treating non-small-cell lung cancer (NSCLC).** **Compounds labelled 24 to 28 are devoid of paclitaxel structure which resulted in significantly lower inhibitory percentages than compound DY-16-43 and nr 23.** **HEK-Blue hNOD2 cells; Lewis lung carcinoma (LLC) tumor-bearing mice.**	(72)
**6.**	**Compound nr 25** 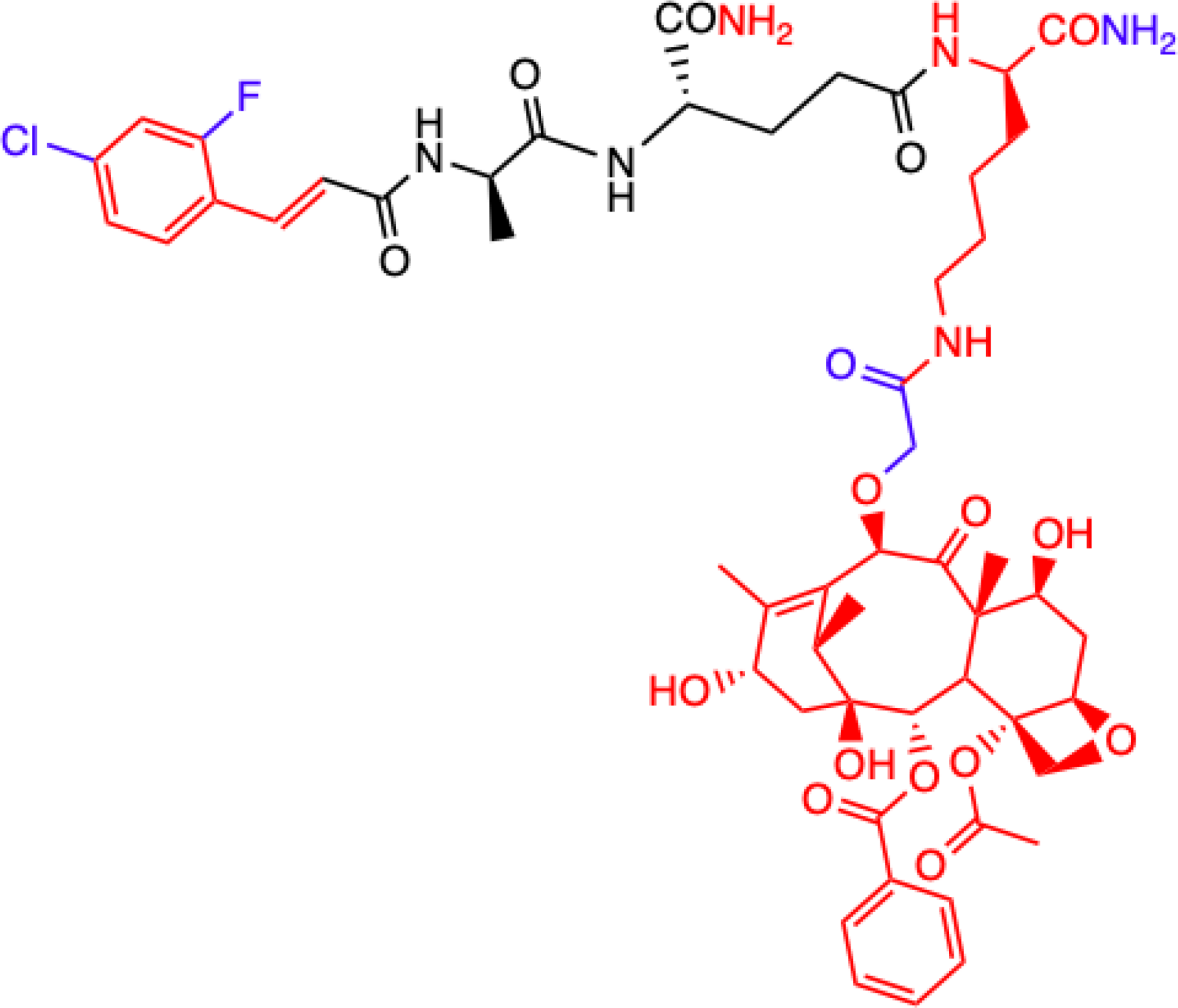 **10-OH substituted 10-DAB, without side chain of PTX; MDA - 4-Cl and 2-F substituted ring**		
**7.**	**Compound nr 26** 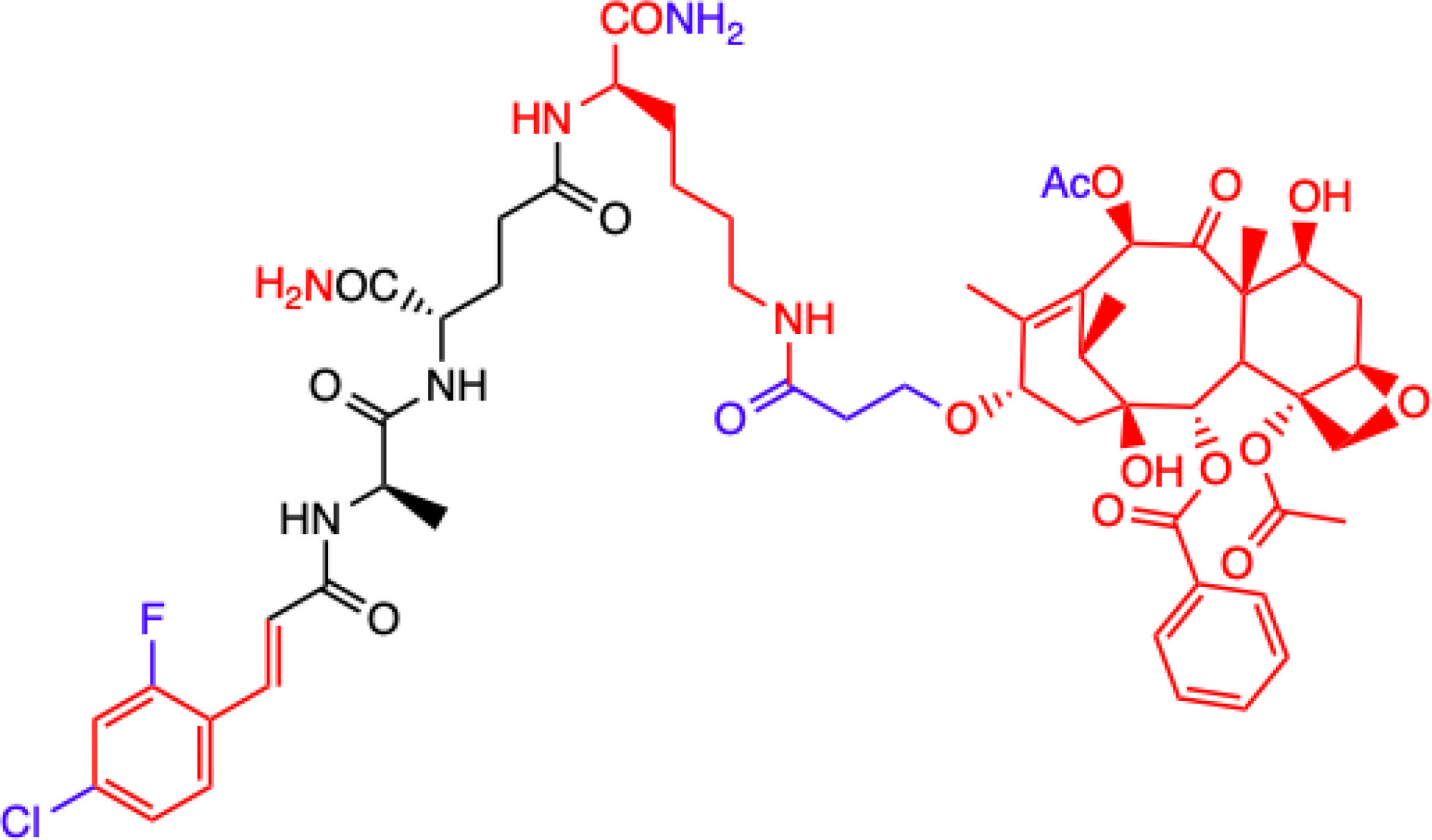 **13-OH substituted 10-OAc-10-DAB, without side chain of PTX; MDA - 4-Cl and 2-F substituted ring**		
**8.**	**Compound nr 27** 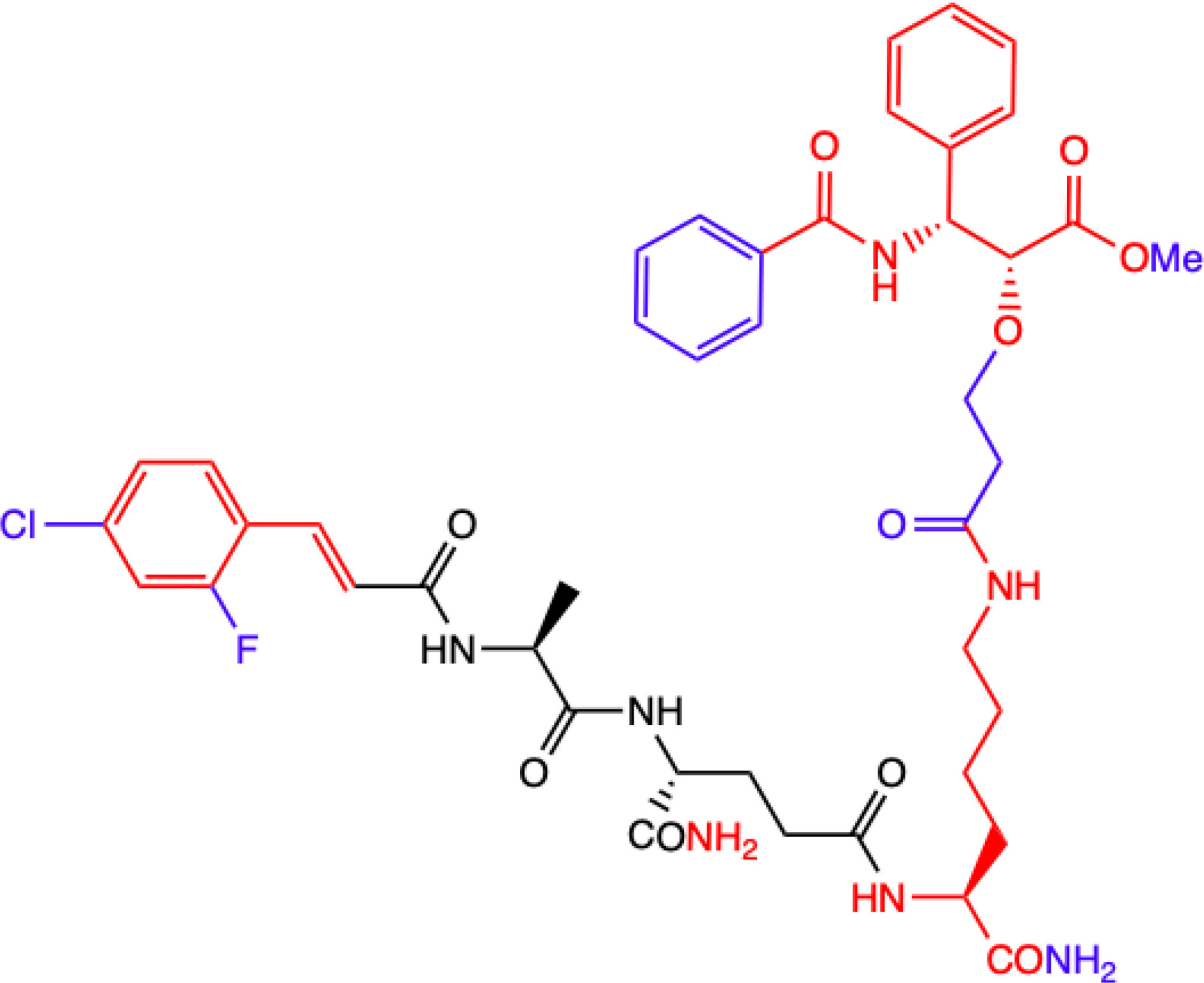 **Side chain of PTX with Me group, without 10-DAB; MDA - 4-Cl and 2-F substituted ring**		
**9.**	**Compound nr 28** 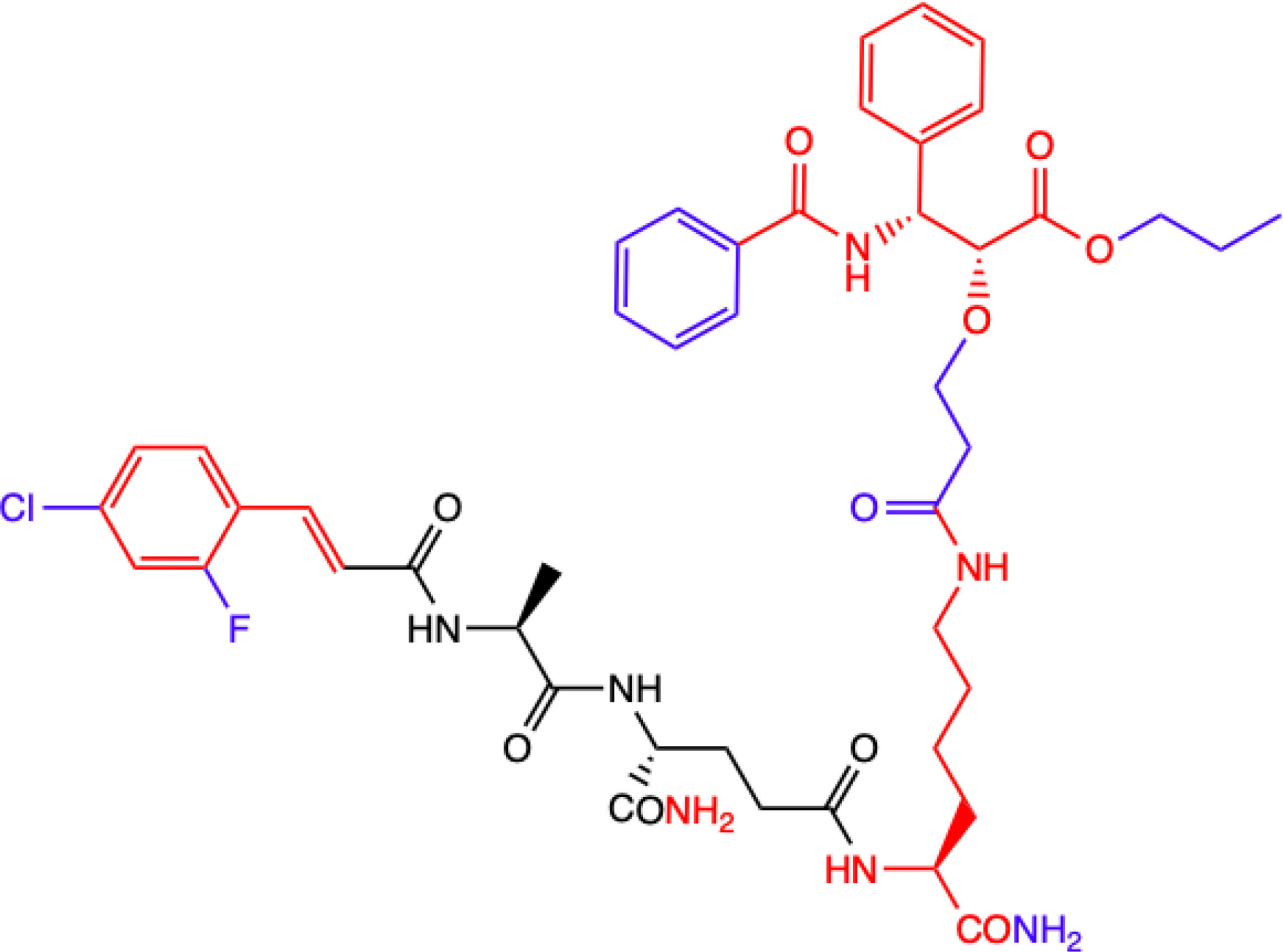 **Side chain of PTX with *n*Bu group, without 10-DAB, MDA - 4-Cl and 2-F substituted ring**		
**10.**	**MDC-405** 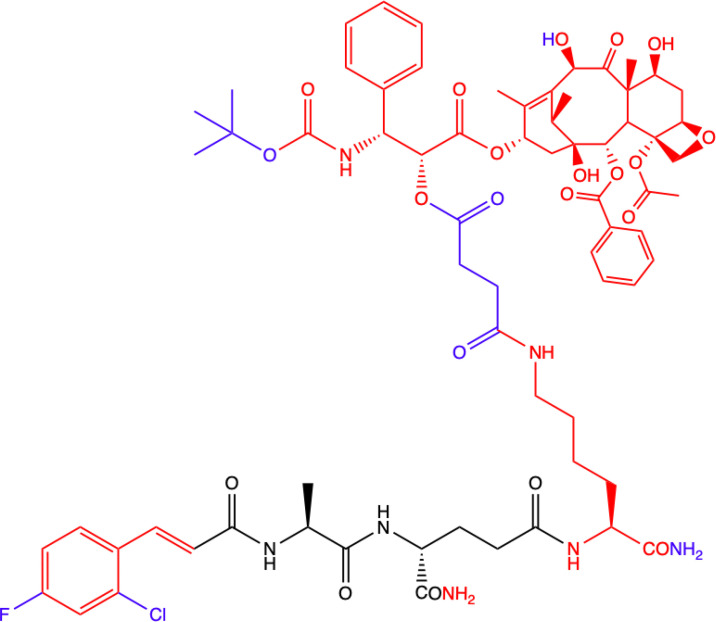 **Docetaxel (PTX analogue) combined with MDA: with CONH_2_ group**	**Excellent pharmacological profile against 4T1 breast tumor growth in mice and against metastasis.** **Unsatisfactory physicochemical properties - primarily very poor water solubility.** **4T1 mammary carcinoma cells**	(73)
**11.**	**Salutaxel** 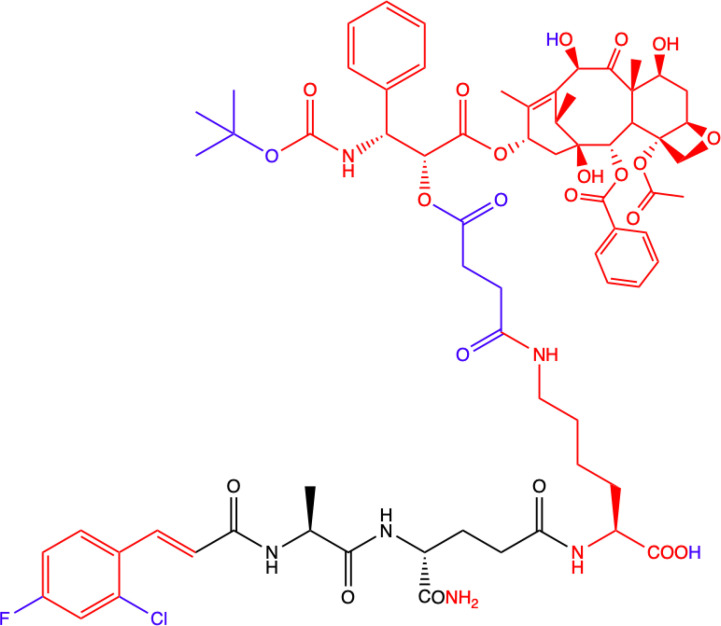 **Docetaxel (PTX analogue) combined with MDA: with COOH group**	**High anticancer efficacy together with satisfactory pharmacological properties.** **Inhibition of the growth of several tumor types *in vivo*.** **Better anticancer properties than docetaxel.** **Human tumor cell line xenograft models: MDA-MB-231 (breast), H1975 (lung), HCT116 (colon), and A549/T (lung for paclitaxel resistance); 4T1 mammary carcinoma cells**	(73)
**12.**	**Compound 8f** 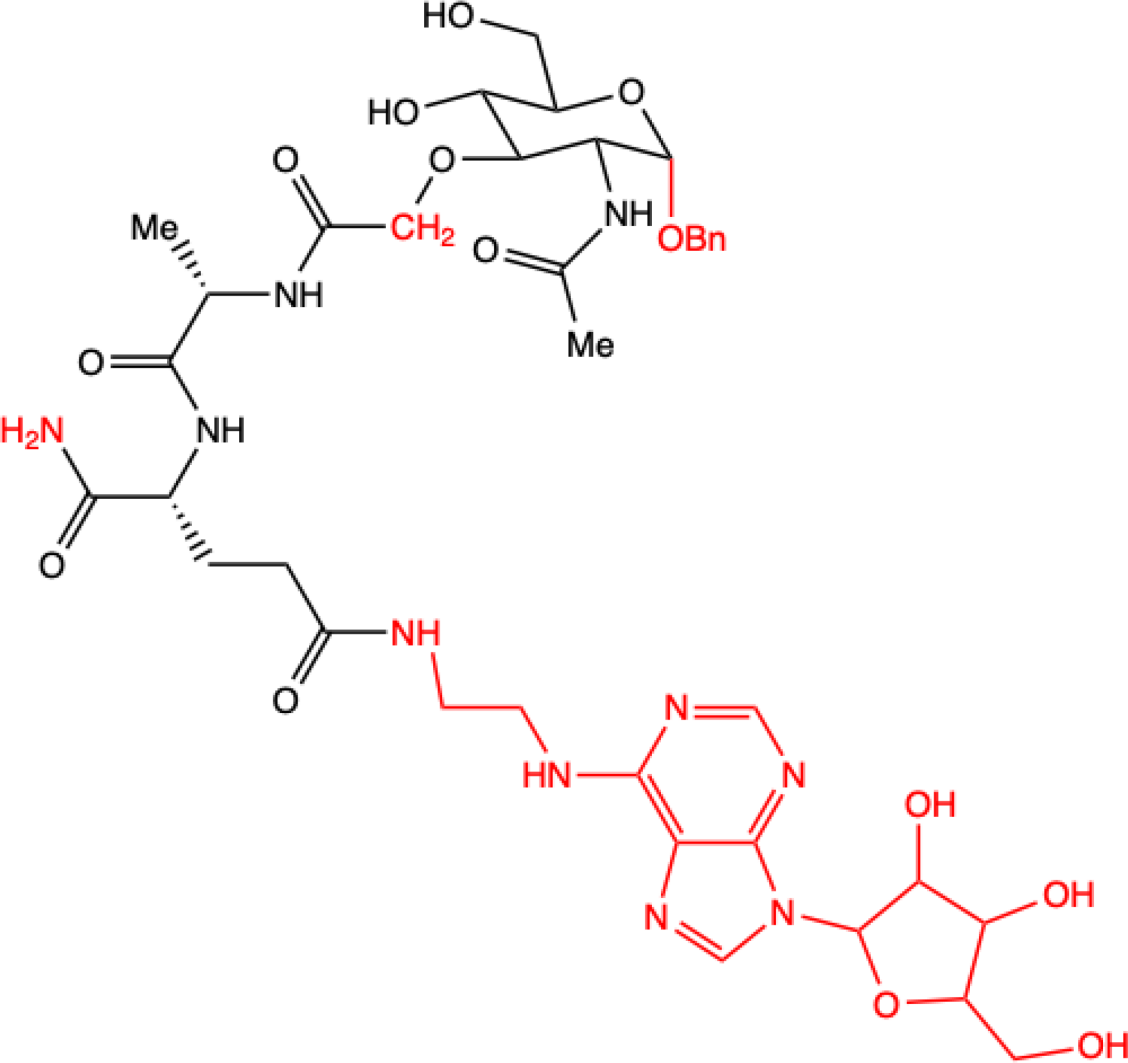 **MDP derivative combined with N^6^-(2-aminoethyl)adenosine**	**Did not show significantly higher antiproliferative activity compared to MDP and nor-MDP but highest index of selectivity.** **Lymphoid cell lines and activated peripheral blood mononuclear cells (PBMC) as *in vitro* model** **Two models of leukemia were used: the human T lymphocyte-based Jurkat cell line and a mouse lymphocytic leukemia L1210.**	(14)
**13.**	**Compound 8g** 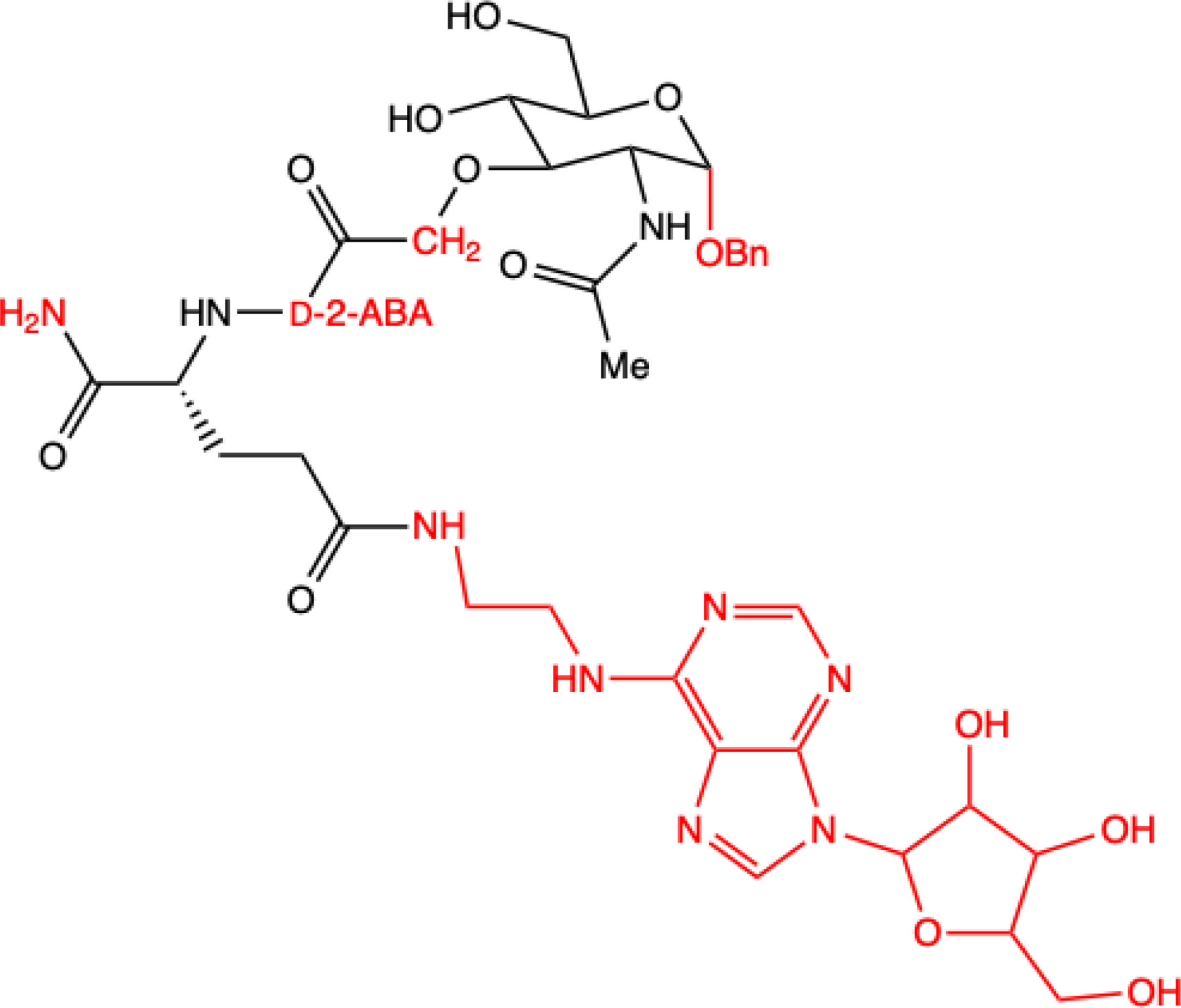 **MDP derivative with abscisic acid (D-2-ABA) combined with N^6^-(2-aminoethyl)adenosine**		
	

**Green**, research model/cell lines; **blue**, sites of change in molecular structures of paclitaxel (PTX) conjugate and MDP analogue; **red**, changes in structure relative to the MDP.

## Conclusion

Since the discovery of the chemical structure and properties of MDP, a huge number of its derivatives have been synthesized. This article describes MDP derivatives that present anticancer properties *in vivo* or cytotoxic activity against cancer cells *in vitro*. Despite significant efforts, at the moment, only two of them, mifamurtide and romurtide, have reached the point of clinical trials that have led to their use for the treatment of patients. This illustrates how difficult it is to achieve adequate anticancer properties together with a favorable pharmacological and pharmacophysical profile to overcome the barrier between preclinical and clinical studies. Difficulties in obtaining satisfactory results of the study are due to the low lipophilicity of the MDP molecule and the occurrence of many adverse effects after administration. It is particularly challenging to find an MDP derivative that will have the desired characteristics when used as an adjuvant. Despite many obstacles, new MDP derivatives are being designed and synthesized to develop and advance the field. This is extremely crucial in the context of the fight against cancer, which is a worldwide problem. By 2030, nearly 13 million people will die from cancer, and about 400,000 children will develop cancer each year.

## Author contributions

EI, II-S contributed to conception and design of the manuscript. EI wrote the first draft of the manuscript. JH wrote sections of the manuscript. KD prepared the chemical nomenclature. All authors contributed to manuscript revision, read, and approved the submitted version.

## Funding

This work was supported by the Grant OPUS18 2019/35/B/NZ7/04212.

## Conflict of interest

The authors declare that the research was conducted in the absence of any commercial or financial relationships that could be construed as a potential conflict of interest.

## Publisher’s note

All claims expressed in this article are solely those of the authors and do not necessarily represent those of their affiliated organizations, or those of the publisher, the editors and the reviewers. Any product that may be evaluated in this article, or claim that may be made by its manufacturer, is not guaranteed or endorsed by the publisher.
